# Mechanism for the Generation of Robust Circadian Oscillations through Ultransensitivity and Differential Binding Affinity

**DOI:** 10.1021/acs.jpcb.1c05915

**Published:** 2021-10-05

**Authors:** Agnish Kumar Behera, Clara del Junco, Suriyanarayanan Vaikuntanathan

**Affiliations:** † Department of Chemistry, University of Chicago, Chicago, Illinois 60637, United States; ‡ The James Franck Institute, University of Chicago, Chicago, Illinois 60637, United States

## Abstract

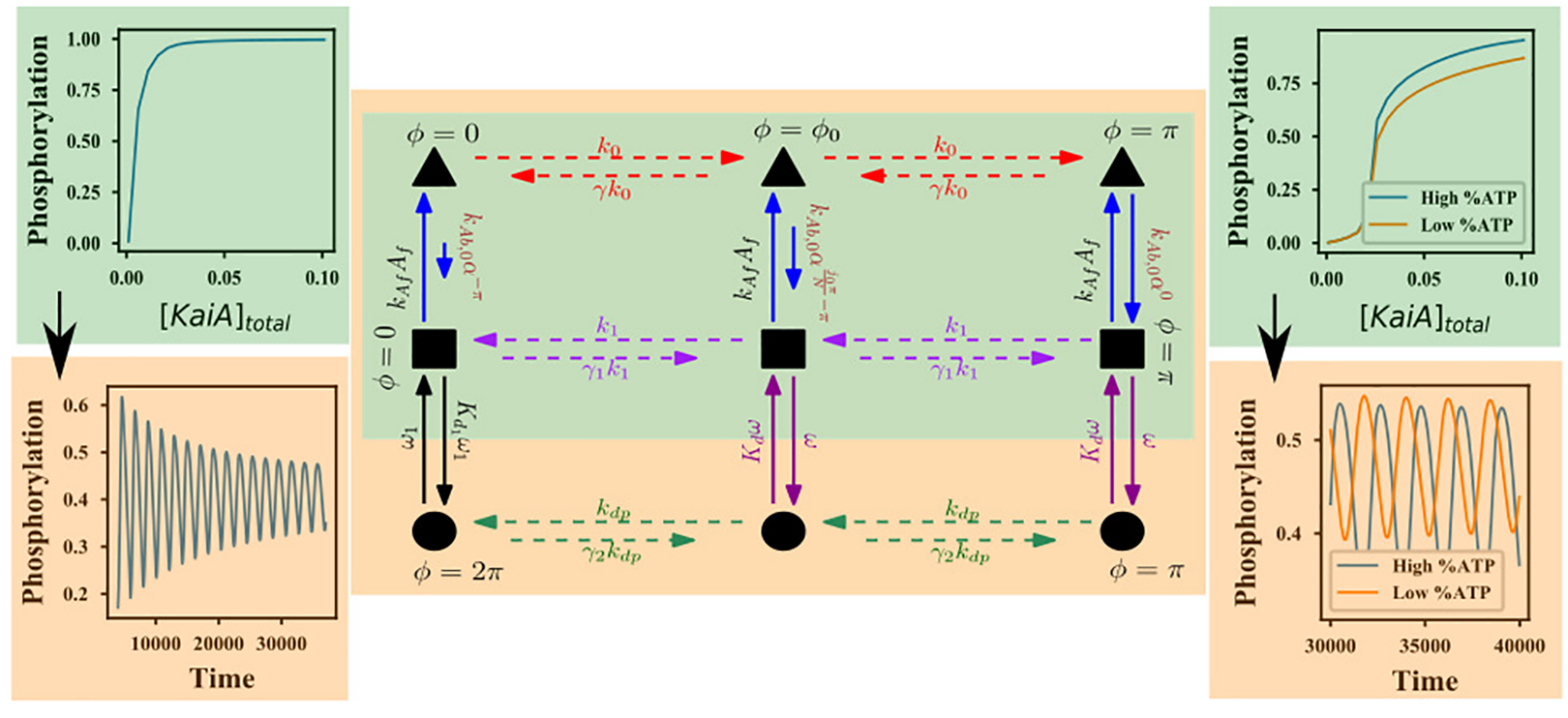

Biochemical circadian rhythm oscillations play an important role in many signaling mechanisms. In this work, we explore some of the biophysical mechanisms responsible for sustaining robust oscillations by constructing a minimal but analytically tractable model of the circadian oscillations in the KaiABC protein system found in the cyanobacteria *S. elongatus*. In particular, our minimal model explicitly accounts for two experimentally characterized biophysical features of the KaiABC protein system, namely, a differential binding affinity and an ultrasensitive response. Our analytical work shows how these mechanisms might be crucial for promoting robust oscillations even in suboptimal nutrient conditions. Our analytical and numerical work also identifies mechanisms by which biological clocks can stably maintain a constant time period under a variety of nutrient conditions. Finally, our work also explores the thermodynamic costs associated with the generation of robust sustained oscillations and shows that the net rate of entropy production alone might not be a good figure of merit to asses the quality of oscillations.

## I Introduction

Most living organisms, ranging from simple single celled organisms like cyanobacteria to multicellular organisms, possess an internal clock which is entrained with the day–night cycle.^1−5^ The fidelity and robustness of this clock are crucial for the well-being and survival of the organism.^6−9^ The time period of the internal clock has, for example, been found to be robust with respect to changes in the temperature, nutrient conditions, and pH.^10−13^ Understanding the biochemical and thermodynamic underpinnings of such robust behavior remains an important challenge given the crucial biological role of the internal clock.

In this paper, we build on recent experimental and modeling work in ref (17) and show how a particular ultrasensitive switch in the KaiABC biochemical circuit can control the quality and robustness of oscillations. In particular, in ref (17), the authors identify a previously underappreciated ultrasensitive response in the phosphorylation levels of the KaiC proteins as the concentration of the KaiA proteins is tuned. The KaiB proteins play no role in this ultrasensitive response. It was postulated in ref (17) that this ultrasensitive switch plays a central role in ensuring robust oscillations. Specifically, the ultrasensitive switch allows the system to exhibit sustained oscillations even at low levels of the energy rich molecule, ATP.^17^ Motivated by this work, we build a minimal Markov state model that provides analytical insight for how an ultrasensitive KaiA–KaiC switch can modulate the quality of oscillations. Our minimal model also allows us to analytically study how another biophysical driving force, namely, the differential affinity of the different forms of KaiC to KaiA,^10,15,19,22^ also controls oscillations. Finally, our minimal Markov state model allows us to comment on the thermodynamic costs associated with setting up robust oscillations in the KaiABC system.

**Figure 1:**
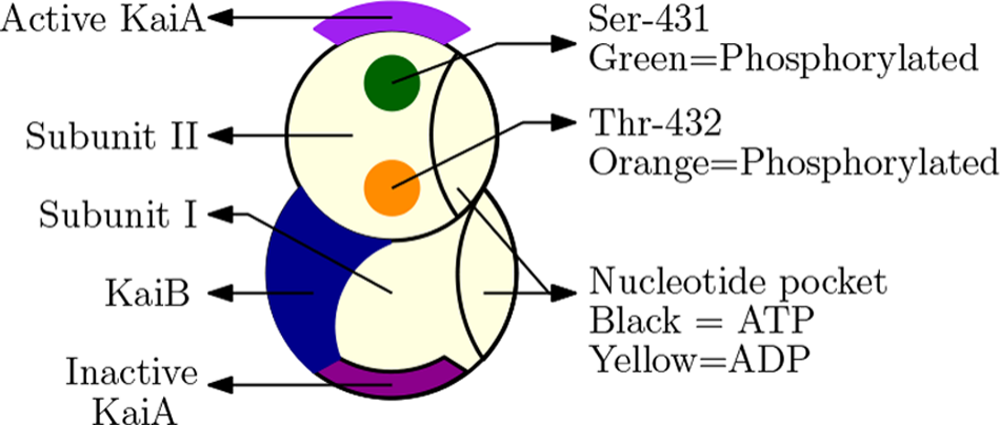
KaiC monomer. The following schematic has been inspired from ref (14). The KaiC protein exists as a hexamer, and each monomer consists of 2 domains, CI and CII. The CII domain has two phosphorylation sites, Ser-431 and Thr-432, a KaiA binding site, and a nucleotide binding site (which binds either ATP or ADP). The CI domain binds to KaiB and helps sequester KaiA. Subsequently, the KaiABC complex will be denoted using ^–/A/B^CI_TP/DP_–^–/A^CII_TP/DP_^U/T/S/D^. Here, TP/DP denotes ATP/ADP attached to the domain: U denotes that none of the sites in CII are phosphorylated, S means that only the serine site is phosphorylated, T means the threonine site is phosphorylated, and D denotes the doubly phosphorylated form. A attached to CI denotes sequestered KaiA; A attached to CII denotes active KaiA acting as an assistant in phosphorylation. B attached to CI implies the inactive form which will start sequestering KaiA.

The KaiABC protein system (see Figure 1) provides a minimal biochemically tractable model to explore the above-mentioned questions. The KaiABC system is found in cyanobacteria *S. elongatus* where it plays the role of regulating the circadian cycle. The KaiABC system consists of three proteins, KaiA, KaiB, and KaiC.^14^ In vitro, the system of KaiABC proteins undergoes sustained oscillations as evidenced by the phosphorylation state of the KaiC protein. These oscillations have been shown to have many of the same robust features as those observed in the circadian oscillations they support in cyanobacteria.^15,16^ The KaiABC model system has been probed in many experimental and theoretical studies.^10,14^ These have elucidated some of the necessary requirements for the generation of sustained oscillations.^10,15,17−21^ Despite these advances, understanding the biochemical and biophysical driving forces that are responsible for sustaining robust oscillations remains an open question.^10,14,16,19,21,22^

The rest of the paper is organized as follows. We first briefly review the salient features of the KaiABC biochemical circuit and then outline our minimal model. This model captures the above-mentioned features of the KaiABC circuit, namely, the differential affinity of KaiC to KaiA binding, and the ultrasensitive response of KaiC phosphorylation levels to changes in KaiA concentration. It also additionally accounts for many other experimentally characterized biophysical forces.^14^ We then write down a stochastic master equation to describe the dynamics of our model. This stochastic master equation is nonlinear in the probability. The nonlinearity is due to the various feedback mechanisms that are necessary for sustaining oscillations. Interestingly, by solving the nonlinear stochastic master equation, we are able to analytically describe the emergence of global oscillations in response to changing the differential affinity.^21^ Our model allows us to obtain approximate analytical solutions that provide qualitative insight into how tuning ultrasensitivity tunes the quality of oscillations. Crucially, our results allow us to elucidate how an ultrasensitive switch can support oscillations even at a lower concentration of ATP. Our results also allow us to explain how the time period of oscillations can be robustly maintained even as the concentration of ATP is tuned, a phenomenon known as affinity compensation. Finally, we comment on the thermodynamic costs associated with sustaining robust oscillations.

## II Methods: KaiABC Oscillator and Model Details

**Figure 2:**
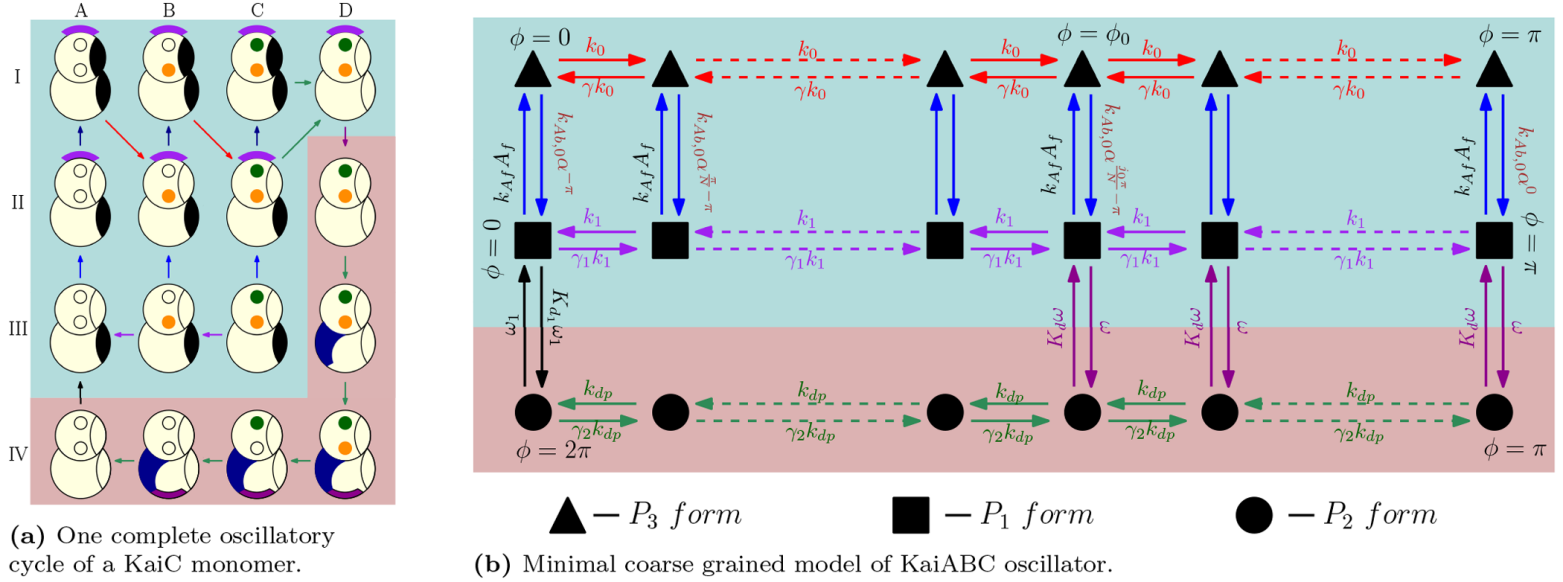
In penel a, rows are labeled I, II, III, IV, and columns are labeled A, B, C, D. In panel a, the colors in the reaction arrows correspond to those in panel b. Active conformations are denoted using a cyan background, and inactive conformations are denoted using a red background. In our model (panel b), the horizontal axis represents the amount of phosphorylation in the system, with ϕ = 0 and ϕ = 2π corresponding to the completely dephosphorylated state and ϕ = π corresponding to the completely phosphorylated hexamer. The phosphorylation function is a linearly increasing function, 0 at ϕ = 0, 1 at ϕ = π, and then symmetrically decreasing from ϕ = π to 2π. Thus, phosphorylation, . Changes in the phosphorylation levels of the KaiC hexamers give rise to oscillations. KaiA binds to KaiC during the ”day” and promotes phosphorylation, whereas at “night”, KaiB binds to KaiC and sequesters KaiA, thus leading to dephosphorylation. The horizontal rungs in all the states correspond to the phosphotransfer reactions and the hydrolysis of ATP accompanying it, i.e., the red arrows between IA → IIB, and IB → IIC, purple arrows between IIIC → IIIA, and green arrows between IVD → IVA in Figure 2a. The ratio of the forward and backward rates is given by, γ, γ_1_, and γ_2_ which are all less than 1, because of the fact that these describe reactions coupled to ATP hydrolysis which are inherently irreversible. In the model, α > 1 is responsible for differential affinity,  corresponds to % ATP, and *k*_1_ helps in tuning ultrasensitivity. Free KaiA, *A*_f_, provides nonlinearity to the system.

The KaiC protein, complexed with KaiA, and KaiB proteins, forms the core of the KaiABC oscillator system. The various possible states of the KaiC protein are described in Figure 1. Our minimal model, described in Figure 2b and inspired by refs (14) and (21) (with additional modifications to include features such as ultrasensitivity), can be viewed as a coarse-grained description of the various biochemical states accessed by the KaiABC protein system.^14^ In the full KaiABC cycle, the KaiABC has two conformations, an active conformation (cyan background in Figure 2) which can phosphorylate the Ser and Thr sites with KaiA as an assistant molecule and an inactive conformation (red background in Figure 2) which sequesters KaiA with the help of KaiB and dephosphorylates the Thr and Ser sites. In our model, the *P*_1_ and *P*_3_ states correspond to the active conformation and *P*_2_ to the inactive conformation.

The various biochemical states of the KaiABC protein are summarized in Figures 1 and 2. Below, we briefly recap the various salient features of the KaiABC oscillatory cycle and explain how they are taken into account in our minimal model.

### II.A Differential Binding of KaiA to KaiC Drives the Phosphorylation Phase

At the beginning of the cycle, most of the KaiC is in the active conformation in the CI_DP_–CII_DP_^U^ form (IIIA in Figure 2a, *P*_1_(0) in Figure 2b), and most of the KaiA is free. Depending on the phosphorylation level of active KaiC, it binds differently with KaiA. At low levels of phosphorylation (IIIA, IIIB), KaiC binds very strongly with KaiA. By constrast, the affinity of KaiA for KaiC is low when the KaiC is in a highly phosphorylated state (IIIC, IIID). This phenomena is termed as a *differential affinity* of KaiC for KaiA dimers.^23^ Our model captures this effect through the parameter α, where α > 1. Specifically, the rates of *P*_1_–*P*_3_ exchange are given by *k*_*A*f_*A*_f_ (where *A*_f_ is the free KaiA concentration) from *P*_1_ to *P*_3_ and by *k*_*A*b,0_α^ϕ−π^ in the reverse direction. As the phosphorylation level increases with ϕ, the term α^ϕ−π^ ensures that the proportion of *P*_1_ (KaiA unbounded) states increases. The extent of differential affinity in our model can be tuned by varying the parameter α. Differential affinity ensures that the unphosphorylated IIIA state is primed for KaiA binding at the start of the phosphorylation cycle. Indeed, KaiA binding to the IIIA state transitions the system into the IIA and IA states. Subsequently, KaiA facilitates rapid exchange of nucleotides which lead to the formation of more ATP bound states and pushes the system toward phosphorylation; i.e., it leads to the formation of CI_TP_–^A^CII_TP_^S^, CI_TP_–^A^CII_TP_^T^, and CI_TP_–^A^CII_TP_^D^ states (IB, IC, and ID states, respectively, in the schematic).

### II.B Dependence of the Kinetic Rates on the ATP Concentration

The concentration of the energy rich molecule, ATP, is an important external condition for the cyanobacteria which affects the KaiABC oscillator. It has been observed that oscillations with almost the same time period are sustained until the % ATP in the system reaches 25% below which oscillations vanish completely.^10^ Here, % ATP . In our model, the concentration of ATP controls the kinetics of the crucial ATP–ADP nucleotide exchange reaction.^14^ Since in our minimal model the reaction corresponding to III(A, B, C) → I(A, B, C) is coarse-grained into *P*_1_(*i*) → *P*_3_(*i*), and since the second step in these reactions, i.e., II(A, B, C) → III(A, B, C), is dependent on % ATP, the % ATP in our model is set by the ratio of the rates connecting the *P*_1_ to the *P*_3_ states:
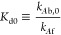
2.1Increasing *K*_d0_ decreases the rate of transitions to the *P*_3_ form and thus corresponds to lower % ATP and vice versa.

### II.C Dynamics of the Dephosphorylation Phase

In the hexamer, the dephosphorylation phase starts even before total phosphorylation of each and every monomer. Specifically, once the number of phosphorylated serine sites becomes larger than the number of threonine sites which are occupied, the KaiA dissociates from the complex, the KaiC transforms into an inactive conformation, and the dephosphorylation phase kicks off. This transition corresponds to ID → IID in the schematic in Figure 2a and to the vertical rungs between *P*_1_ and *P*_2_ states that are colored magenta in our model in Figure 2b.

The dephosphorylation phase (IVD → IVA) is relatively simple. It does not require KaiA as an assistant molecule for the reactions. When the proportion of doubly phosphorylated KaiC (ID, IID) is high, KaiB binding to the CI domain of KaiC is triggered, IID → IIID. In our model, the KaiB binding to KaiC is taken into account implicitly during the transition from *P*_1_ to *P*_2_ states. KaiB bound KaiC, ^B^CI_DP_–CII_DP_^D^ (IIID), sequesters KaiA, i.e., binds to KaiA and makes it unavailable for active use. This is taken into account through the parameter ϵ_seq_ in our model which reduces the free KaiA in the system by an amount ϵ_seq_∑*P*_2_. The dephosphorylation proceeds through the serine sites and then the threonine sites. Dephosphorylation reactions occur through phosphotransfer.^22^ This corresponds to the system moving through the *P*_2_ states in our model. As the reactions reach the completely dephosphorylated state ^AB^CI_DP_–CII_DP_^U^ (IVB), the KaiABC complex starts dissociating into KaiC and KaiB and releasing free KaiA into the system (IVB → IVA). The connection between *P*_2_(0) and *P*_1_(0) in our model takes this dissociation step. This prepares the system for the next cycle.

### II.D Ultrasensitive Response of KaiC Phosphorylation to KaiA Concentration

**Figure 3:**
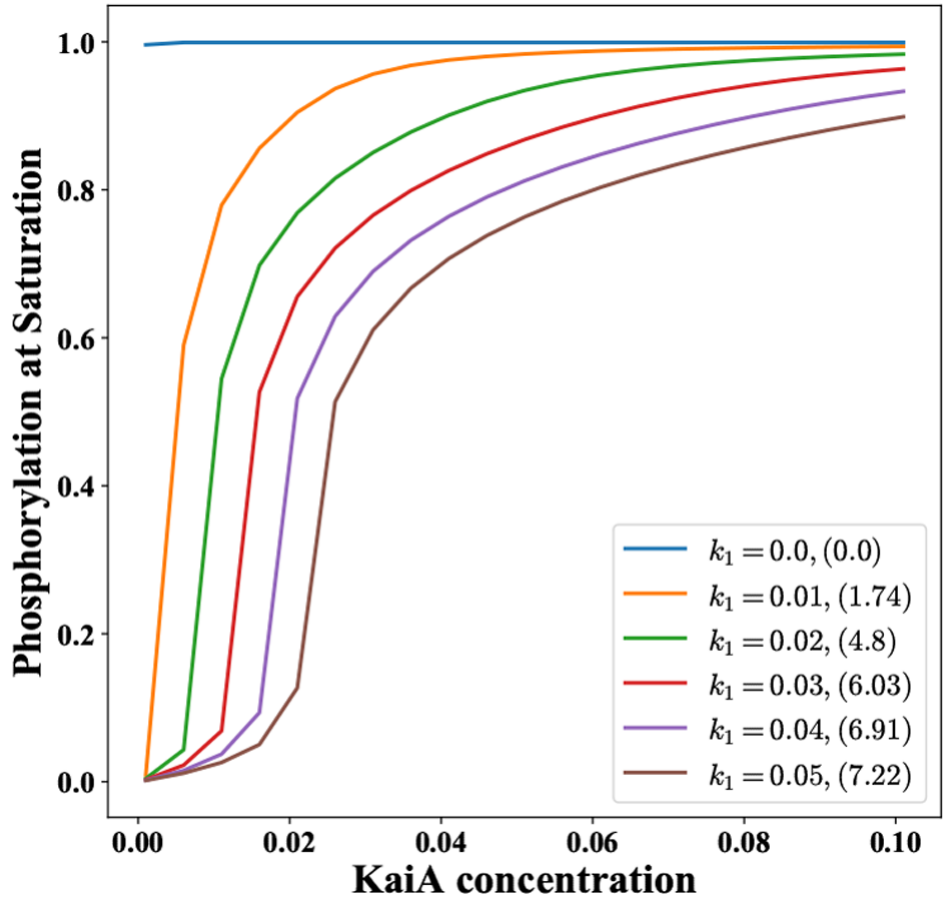
Ultrasensitive response in phosphorylation of KaiC with regard to the total KaiA concentration for *K*_d0_ = 10 and α = 10. The values in the bracket are the Hill coefficients for the response curves (calculated using the method of relative amplification^26^). Values of other parameters are given in Table S2. These kinetics are in the absence of KaiB and *P*_2_ states (ω = ω_1_ = 0); i.e., they represent only the active form of KaiC in Figure 2b. Thus, there are no oscillations, and the system always settles into a final steady state.

It has been experimentally observed that, in the absence of KaiB in the system, KaiC shows an ultrasensitive response in phosphorylation to KaiA concentration in the system; i.e., the phosphorylation level of the KaiC hexamers changes rapidly within a very narrow range of total KaiA concentration.^10,17^ This ultrasensitivity was speculated to be an important prerequisite for sustaining robust oscillations, particularly in conditions wherein the concentration of the energy rich molecule, ATP, is low. Our model captures the ultrasensitive response observed in ref (17) and described in Section II, through the introduction of the dephosphorylation rate *k*_1_ (see Figure 3). Indeed, a standard way to obtain an ultrasensitive response is through the action of two antagonistic enzymes working at saturation.^24,25^ Under such conditions, the response of the system changes rapidly over a very narrow range of the enzyme concentration. In the KaiABC system, the roles of the antagonistic enzymes are played by KaiA, which acts as a kinase phosphorylating KaiC and KaiC, which acts as its own phosphatase dephosphorylating itself.^10,22^

The rate *k*_1_ in our model captures this dephosphorylation. Tuning dephosphorylation rates by increasing *k*_1_ leads to competition between phosphorylation in the *P*_3_ states and dephosphorylation in the *P*_1_ states. In the absence of KaiB, which corresponds to setting ω = ω_1_ = 0 in our model, we consequently observe an ultrasensitive response of phosphorylation level of KaiC to changes in the KaiA concentration (Figure 3).

### II.E Dependence of the Kinetic Rates on the KaiA Concentration

As has been described above, the rates of transition between the *P*_1_ and *P*_3_ states in our minimal model depend on the concentration of free KaiA, *A*_f_. The amount of free KaiA in turn depends on the concentrations of the *P*_3_ and *P*_2_ states since the KaiC complex is bound to KaiA in these states. Subsequently, . As the amount of *P*_3_ and *P*_2_ states increases, the free KaiA concentration decreases. This step gives rise to nonlinearity in the system.

## III Results: Role of Differential Affinity and Ultrasensitivity. Insights from an Analytical Treatment of the Nonlinear Fokker–Planck Equations

Our minimal model described in Figure 2b and Section II can be represented mathematically using a nonlinear Fokker–Planck equation,, where *P⃗* is the probability vector of all the states (*P*_1_, *P*_2_, *P*_3_), and **W** = **W**(*P⃗*) is the rate matrix that is dependent on the state of the system. The nonlinear Fokker–Planck equation is described in full detail in the Supporting Information, Section S1.

If there were no nonlinearity in the Fokker–Planck equation, the Perron–Fobenius theorem would have ensured that the Fokker–Planck equation has a stable time-independent steady-state solution. The oscillatory solutions of the rate matrix decay with time as they have eigenvalues with a negative real part. Due to the nonlinearity in the Fokker–Planck equation in the Supporting Information, eq S1.5, time-dependent oscillatory steady-state solutions may be possible.

In this work, we focus on how the solutions of the Fokker–Planck equation change as two specific parameters, namely, α controlling the differential affinity and *k*_1_ controlling the ultransensitivity, are varied. In particular, we analytically show how the system can be made to transition from a time-independent steady state, where it cannot function as a biological clock, to a time-dependent steady state, where it can function as a biological clock, as the differential affinity parameter α is tuned. For the case where the ultrasensitivity parameter *k*_1_ is tuned, we take inspiration from our solution from tuning α and obtain an approximate solution. Our approximate analytical arguments provide insight into how ultrasensitivity also supports the functioning of the biological clock.

Finally, as has been reported in many experimental and theoretical studies,^10,14,16^ oscillations are affected by the concentration of % ATP in the system. In particular, it has been found that the KaiABC system cannot sustain oscillations below a critical ATP concentration. In the next section, we will use our minimal model to show how stronger differential affinity and a better ultrasensitive switch can in fact sustain oscillations even at lower ATP concentrations.^17^

**Figure 4:**
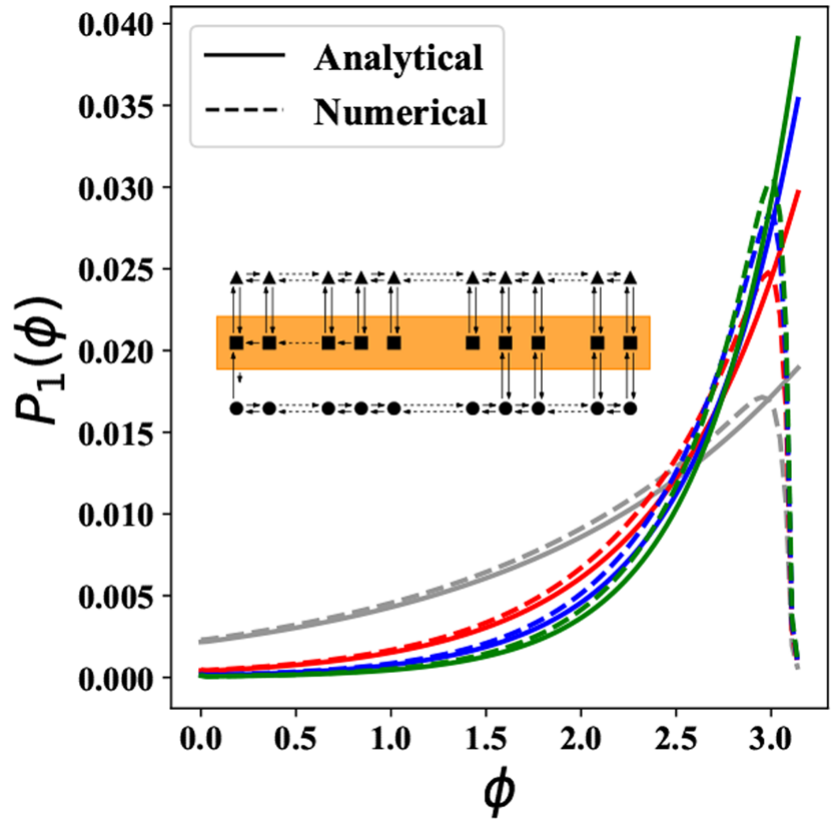
Comparison between numerical and analytical results for the time-independent solution of *P*_1_ states (eq 3.2) for different α’s. The figure in the inset is a representation of the Markov state network with the *P*_1_ states highlighted. In the main figure, gray corresponds to α = 2, red to α = 4, blue to α = 6, and green to α = 8.

We begin our analytical treatment by first considering the case where *k*_1_ = 0, i.e., in a model devoid of ultrasensitivity. In this case, a time-independent solution for the nonlinear Fokker–Planck equation can be obtained in the limit when ϵ_seq_ = 0 and ϕ_0_ = π. ϵ_seq_ = 0 corresponds to the absence of KaiA sequestration by KaiB bound KaiC states. ϕ_0_ = π means that the dephosphorylation phase starts only after all the KaiC species have become doubly phosphorylated. Our analytical derivation is discussed in detail in Supporting Information, Section S2A, and leads to the following solutions (Figure 4).
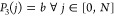
3.1
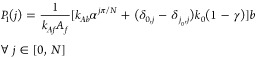
3.2
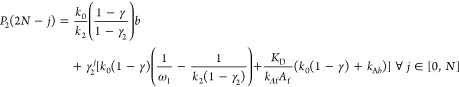
3.3where *b* = *P*_3_(0) can be obtained by solving a quadratic equation as mentioned in the Supporting Information, Section S2, . Even when ϕ_0_ < π, our solution gives a very good approximation if we set *P*_1_(*j*) ≈ *P*_2_(2*N* – *j*) ≈ *P*_3_(*j*) ≈ 0 ∀*j* ∈ [*j*_0_, *N*].

**Figure 5:**
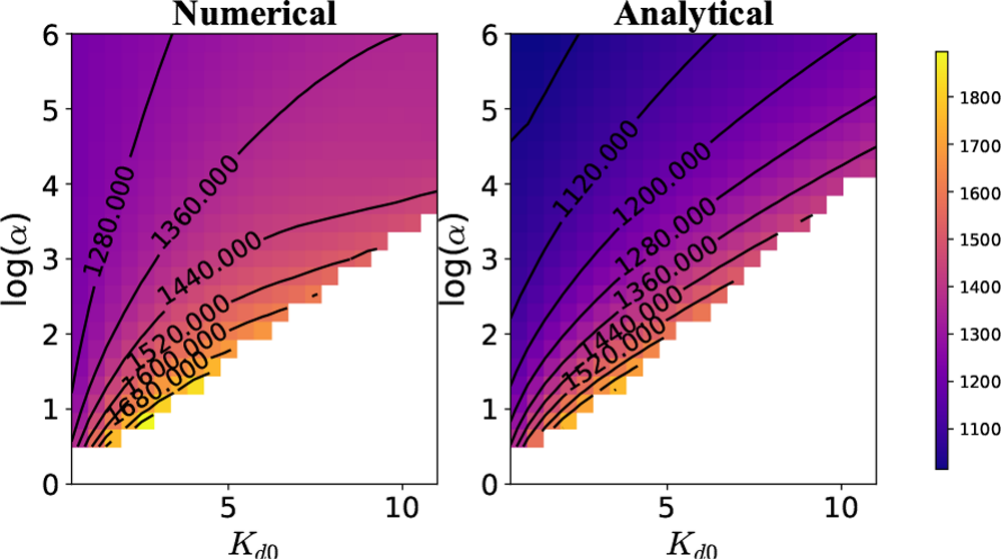
Time period of oscillations for various α and *K*_d0_, i.e., at varying levels of differential affinity and % ATP. *k*_1_ = 0. Other parameters are given in Table S1. Since *k*_1_ = 0, there is no effect of ultrasensitivity. The figure on the left represents time periods calculated by numerically simulating the FPEs. The figure on the right represents the time periods which were calculated from the imaginary part of the maximum positive eigenvalue of the instability matrix **W**, for small perturbations around the steady-state probability distribution. As can be seen, the analytical solution provides us with a good approximation of the time period as well as the critical α at which oscillations take place for different *K*_d0_ values. The contours in the figure are for the time period of the oscillations.

As α is increased, this time-independent state becomes unstable giving rise to a oscillatory state. As described in the Supporting Information, Section S3, a linear stability analysis can be performed around the steady state of the system, , to characterize this instability. The linear stability analysis has been detailed in the Supporting Information, Section S3A. This analysis correctly predicts the observed oscillatory behavior. Indeed, in Figure 5, we show that the analytical estimate of the time period of oscillations provides a very good description of the actual observed oscillation periods.

**Figure 6:**
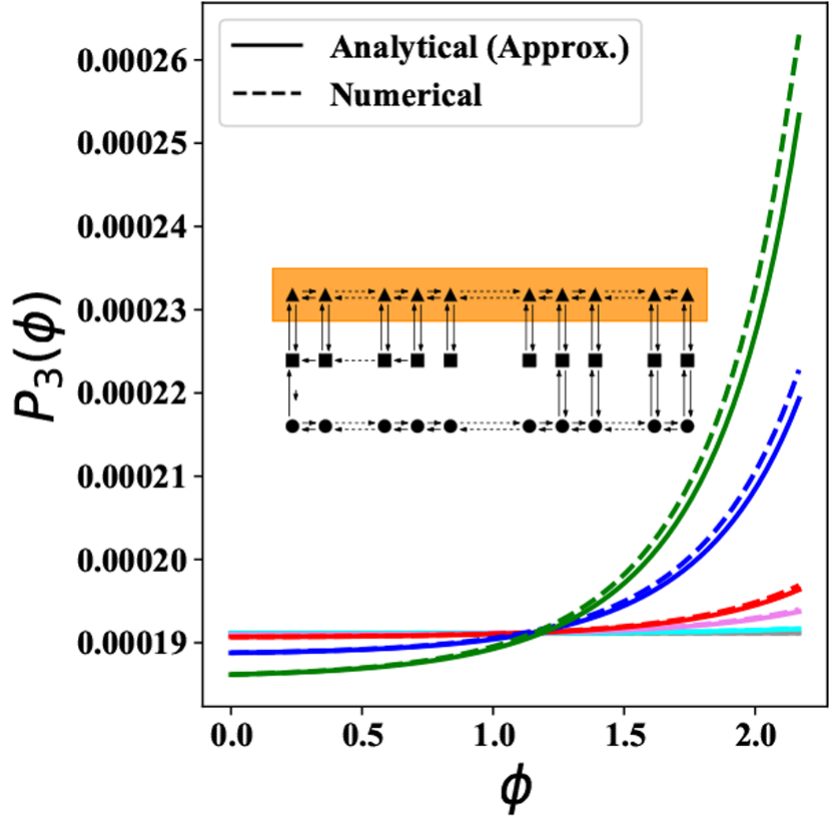
Comparison between numerical and approximate analytical results for the time-independent solution of *P*_3_ states for the case when *k*_1_ ≠ 0 (eq 3.4). The figure in the inset represents the Markov state network with the *P*_3_ states highlighted. In the main figure, gray corresponds to *k*_1_ = 0, cyan to *k*_1_ = 10^–4^, violet to *k*_1_ = 5 × 10^–4^, red to *k*_1_ = 10^–3^, blue to *k*_1_ = 5 × 10^–3^, green to *k*_1_ = 10^–2^.

In the case of *k*_1_ ≠ 0, only an approximate solution for the time-independent steady state can be obtained. In order to obtain this approximate solution, we take inspiration from the solution for the case when *k*_1_ = 0 and assume *k*_*A*f_*A*_f_*P*_1_(ϕ) = *k*_*A*b0_α^ϕ−π^*P*_3_(ϕ) for ϕ ∈ [0, ϕ_0_] (along the *P*_1_–*P*_3_ connections in Figure 2b) and *P*_1_(ϕ) ≈ 0 ≈ *P*_3_(ϕ) for ϕ > ϕ_0_. This assumption is supported by numerical evidence. Under this assumption, we obtain
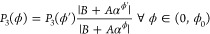
3.4
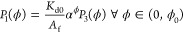
3.5
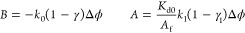
3.6Here, *P*_3_(0) can be obtained numerically, and ϕ_0_ denotes the place where *P*_1_–*P*_2_ connections start in Figure 2b. This is described in more detail in Supporting Information, Section S2B. Figure 6 shows a comparison between the numerically obtained steady state with the one constructed using our approximate solution. We also provide approximate analytical arguments to show how a linear instability analysis can again be used to characterize the onset of oscillations as *k*_1_ is tuned. The Gershgorin circle theorem provides us with a way to understand where we can find the eigenvalues of any matrix. As *k*_1_ is tuned, the negative off-diagonal elements of the rate matrix **W** increase in magnitude, as do the radii of the Gershgorin discs (see Figure S7), because, for any transition rate matrix, **M**, *∑*_*i*_*M*_*ij*_ = 0. In effect, the Gershgorin discs have a finite area protruding into the positive half-plane. With higher *k*_1_, this area increases; thus, there is a higher chance of finding eigenvalues in the positive half-plane. These arguments are explained in more detail in the Supporting Information, Section S3A.

In the next section, we build on these results and show how ultrasensitivity and differential affinity can support oscillations even at a lower ATP concentration. We also use the insight from these analytical arguments to explain how the time period can be stably maintained in a variety of ATP concentrations, a phenomenon known as affinity compensation. Finally, using our minimal model, we also comment on the thermodynamic costs associated with maintaining oscillations.

## IV Discussion

### IV.A Increasing Differential Affinity Leads to Oscillations at Low % ATP

It has been numerically shown previously in ref (21) that oscillations in a model system similar to ours can be obtained by increasing the value of α, i.e., by improving the differential affinity. α controls the rate of reaction between *P*_1_ and *P*_3_ states in Figure 2b. Our analytical results explain this numerical observation. Further, our analytical results at *k*_1_ = 0 also help predict the required interplay between α and the ATP concentration in order for oscillations to be sustained. Specifically, we find that, at *k*_1_ = 0, a higher value of α is required for oscillations to take place at higher *K*_d0_ (or a lower ATP concentration). In Figure 8, we provide estimates of how the critical value of α changes as a function of the *K*_d0_. Our analytical estimates agree very well with those obtained from the numerical calculations.

**Figure 7:**
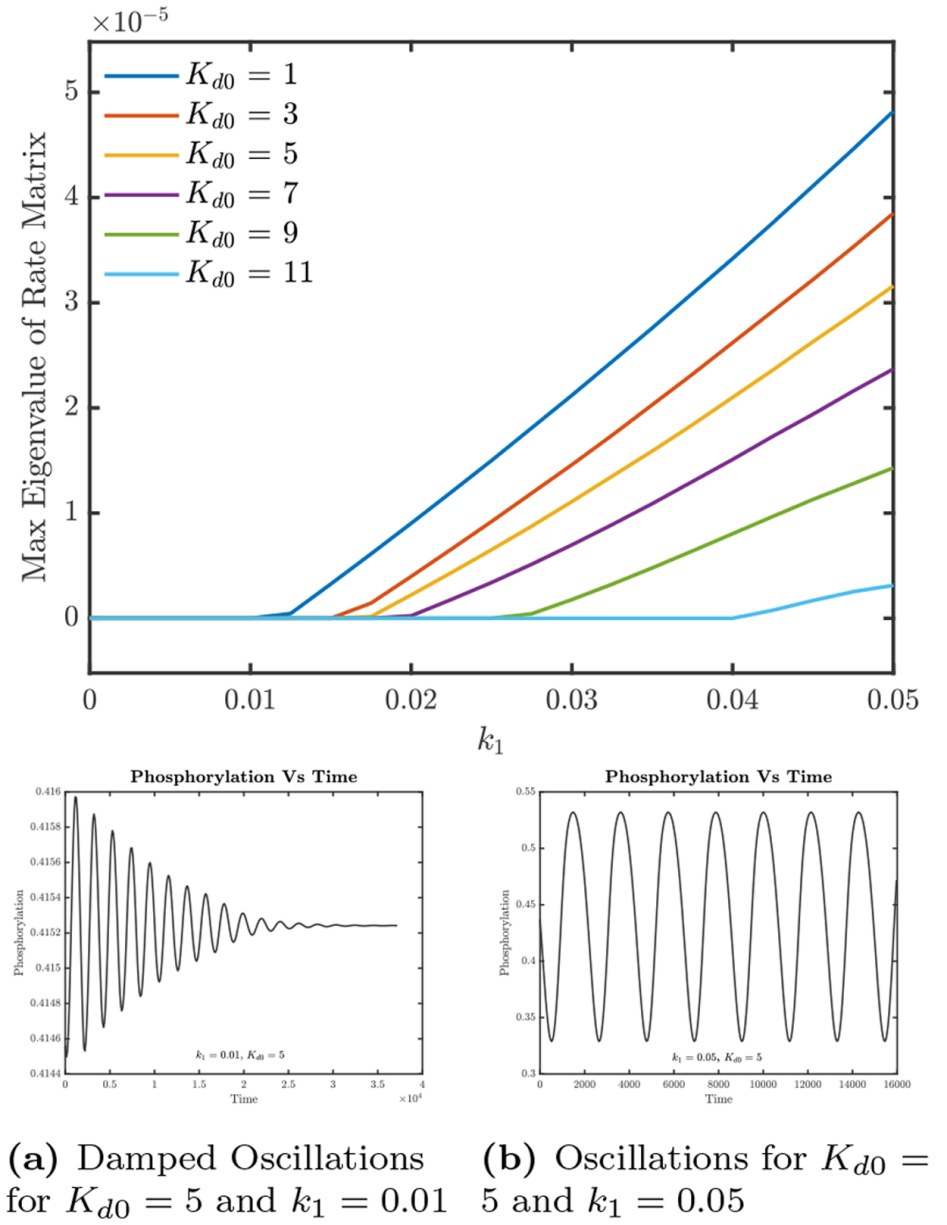
Instability leading to oscillations when changing *k*_1_. The *y*-axis denotes the maximum eigenvalue of the rate matrix **W** for the perturbations (refer to Supporting Information). The presence of a positive eigenvalue denotes that the time-independent steady state is unstable. α = 10, and the other parameter values are listed in Table S2.

**Figure 8:**
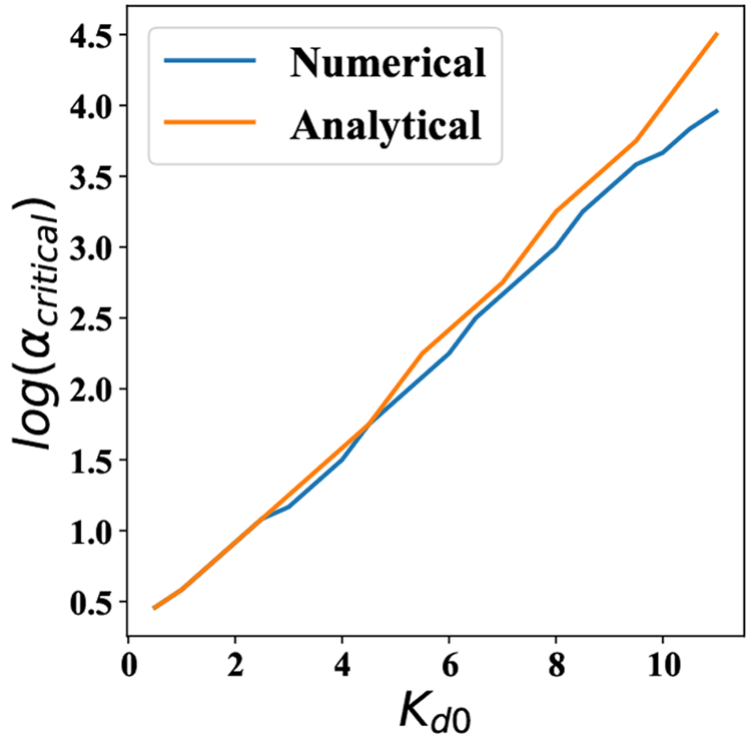
Value of α required for the onset of oscillations as a function of *K*_d0_. Estimates have been obtained both from our theory and from numerical simulations. We set *k*_1_ = 0 for these calculations.

### IV.B Improving the Ultrasensitive Response Leads to Oscillations at Lower % ATP and Fixed Differential Affinity

**Figure 9:**
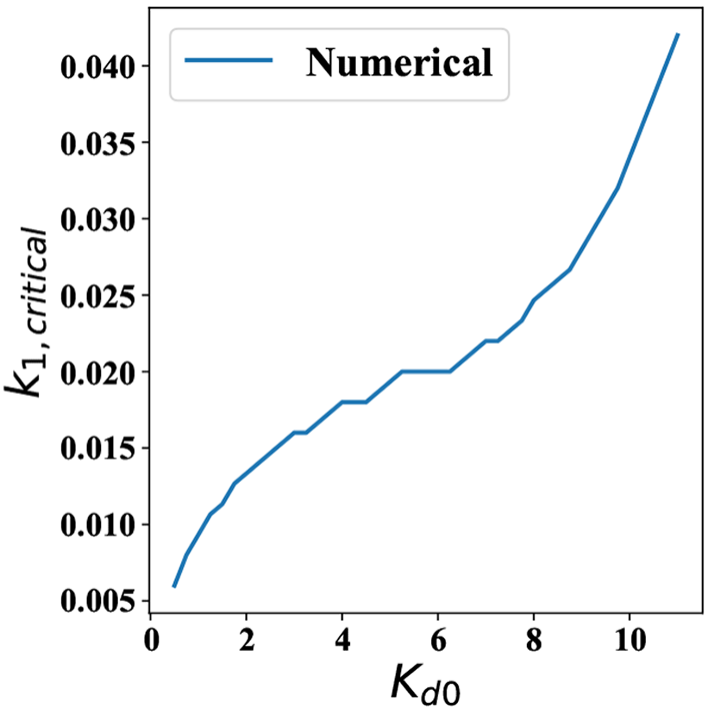
Value of *k*_1_ required for the onset of oscillations as a function of *K*_d0_. Since *k*_1_ ≠ 0 is only approximately tractable analytically, we have only plotted estimates from numerical simulations.

As mentioned in Section II, it has been speculated that ultrasensitivity plays an important role in sustaining oscillations at low % ATP conditions. Our minimal model captures this role played by ultransensitivity. Indeed, we find that, at a higher value of *k*_1_, corresponding to a sharper ultransensitive response (Figure 3), oscillations can be sustained for a larger *K*_d0_ (or a smaller ATP concentration). We describe this trade-off in Figures 7 and 9.

Our analytical analysis also allows us to provide a phenomenological understanding of the role played by the ultransensitive switch. Ultrasensitivity offers coherence to the traveling wave packet of phosphorylation at the start of every new cycle of oscillation. Phosphorylation is halted until a critical amount of KaiA is present in the system. Just before the beginning of every new phosphorylation cycle, most of the KaiA is sequestered by the *P*_2_ states. Only after a certain amount of KaiA is freed from *P*_2_ states can the phosphorylation reactions in the *P*_3_ states start again. This leads to a build-up of probability density near *P*_2_(2π) and *P*_1_(0) before the start of every cycle and provides coherence to the system, and oscillations can be sustained.

### IV.C Metabolic Compensation of Time Period: Insights from the Minimal Markov State Model

**Figure 10:**
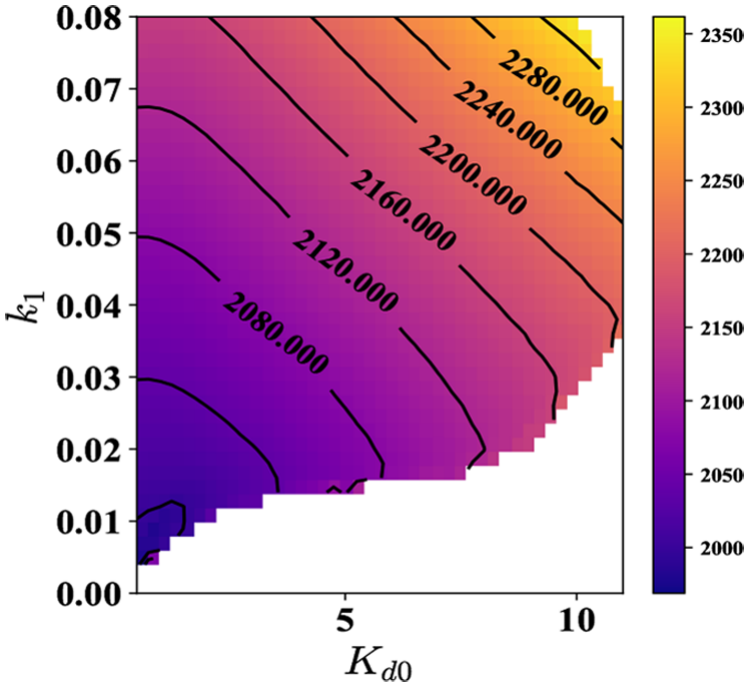
Time period of oscillations for various *K*_d0_ and *k*_1_ values, i.e., at different levels of % ATP and ultrasensitivity. The white region denotes the parameter space which does not support oscillations. This is also supported by the plot for the amplitude of oscillations, Figure 11. In order to have oscillations at higher values of *K*_d0_, the system requires a higher value of *k*_1_. The contours in the figure are for the time period of oscillations.

**Figure 11:**
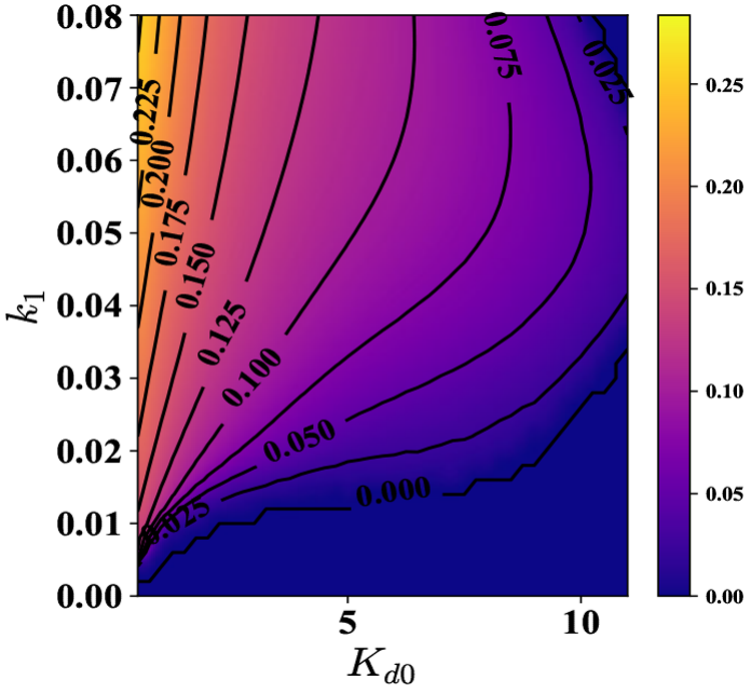
Amplitude of oscillations as a function of *K*_d0_ and *k*_1_ at α = 10 and parameters given in Supporting Information, Section S2. The contours in the figure are for the amplitude of oscillations.

**Figure 12:**
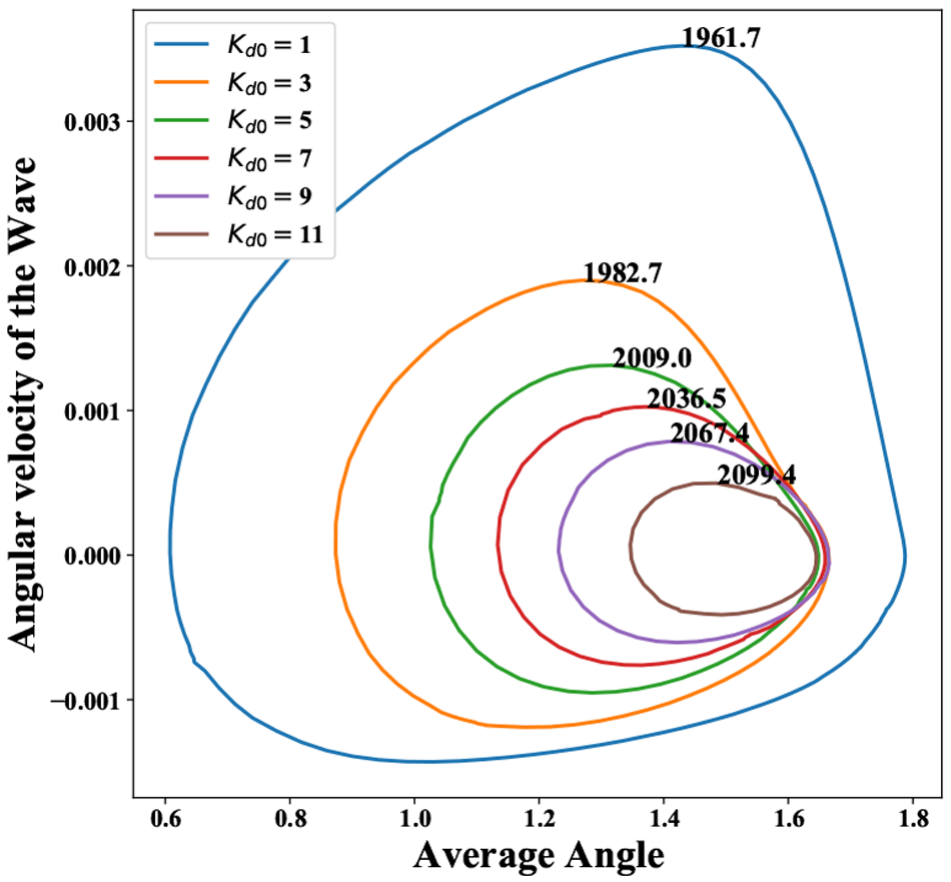
Velocity of phosphorylation wavepacket as a function of average angle for *k*_1_ = 0.05, with various *K*_d0_’s and other parameters as given in Table S2. Here, the average angle ⟨ϕ⟩ = *∑*_ϕ_*ϕP*(ϕ), and velocity . The time period of oscillation for the different cycles is denoted along the curves.

One of the most important features of the KaiABC oscillator is that the time periods of the oscillations are robust to changes in the % ATP in the system, a phenomenon known as metabolic compensation. Our model shows a similar behavior. Upon increasing *K*_d0_, the time period increases, changing by 10% for an increase from *K*_d0_ = 1 to 11 (see Figures 10, 11, and 12). At *K*_d0_ > 11, oscillations are not supported. This is analogous to losing oscillations when % ATP is below 20% ATP in the real system.^10,14^

Our minimal model helps provide a simple phenomenological explanation of affinity compensation. In the regime where our model allows oscillations, the speed of the waveform as it traverses the top *P*_1_–*P*_3_ rungs in Figure 2b from regions of lower ϕ to regions of higher ϕ can be shown to be  through a first-passage time analysis (outlined in Supporting Information, Section S4). Thus, it is expected to decrease with *K*_d0_. Simultaneously, 1/*K*_d0_ ≡ *k*_*A*f_/*k*_*A*b,0_ can be expected to control the relative occupancy of the *P*_1_–*P*_3_ states, and the transitions in the *P*_3_ states promote the probability flux toward regions of higher ϕ. Thus, with increasing 1/*K*_d0_, the waveform can be expected to traverse more of the large ϕ states in the *P*_3_ rung before transitioning to the *P*_1_ and then eventually to the *P*_2_ states as it restarts the oscillation. Hence, at higher 1/*K*_d0_ or higher % ATP, the system traverses a larger orbit as described in the “angle-angular velocity” phase space (Figure 12). This is analogous to shifting in the trough and crest in the phosphorylation oscillations observed in the KaiABC system.^10^ Together, these effects make the time period of oscillations relatively insensitive to % ATP levels (Figure 12). In this way, the KaiABC system can accomplish affinity compensation and maintain a relatively constant time period.

### IV.D Thermodynamic Costs of Setting Up Oscillations

**Figure 13:**
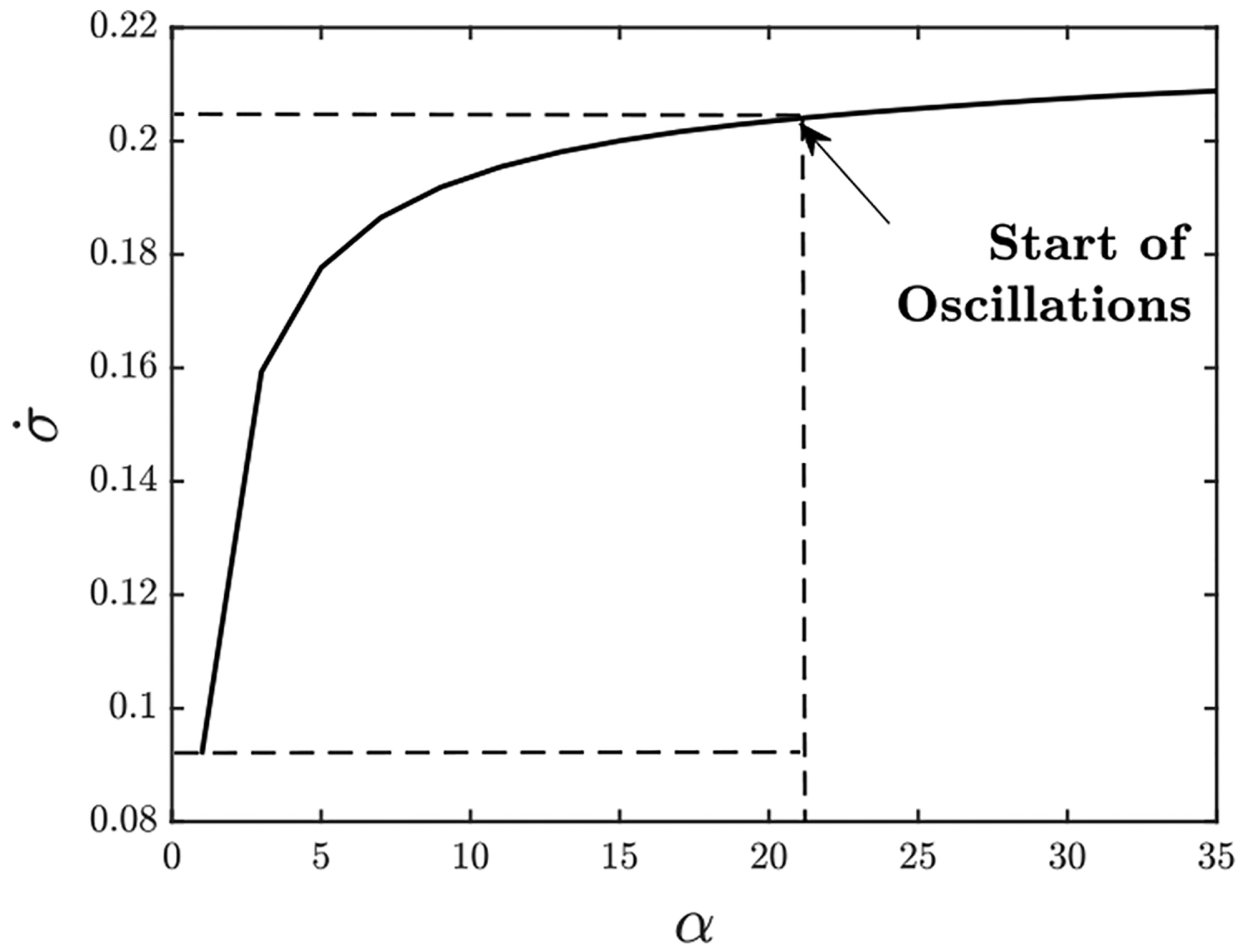
Entropy production rate vs α for *K*_d0_ = 5, *k*_1_ = 0, and other parameters given in Table S1. Oscillations start at α = 21. α = 1 corresponds to the absence of differential affinity. In order to have oscillations, an additional 0.113 units of energy are required. This energy goes into building coherence among the KaiABC oscillator population^21^

**Figure 14:**
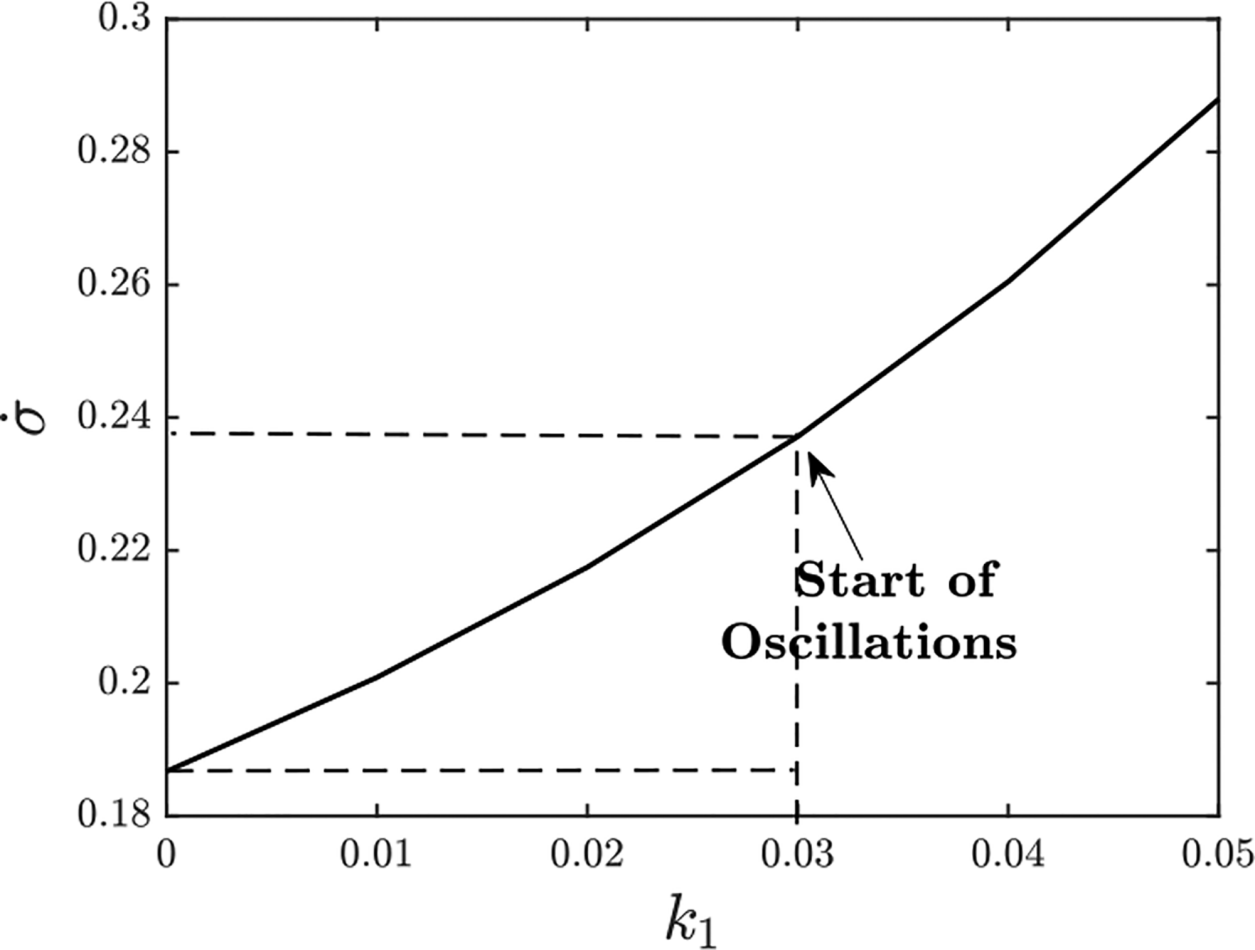
Entropy production rate vs *k*_1_ for α = 10, *K*_d0_ = 8 and other parameters given in Table S2. Unlike the case with changing α in Figure 13 where the entropy production plateaus very quickly with increasing α, in this case, the entropy production increases almost linearly with increasing *k*_1_. As expected, decreasing *K*_d0_ and increasing *k*_1_ lead to a higher dissipation of energy. Oscillations start at *k*_1_ = 0.03. *k*_1_ = 0 corresponds to the absence of ultrasensitivity in the system. An additional 0.052 units of energy are dissipated in order to have oscillations. This additional energy goes into improving the ultrasensitive response of the system, eventually leading to coherence.

Finally, the stochastic thermodynamics of our minimal model can be readily probed. The total steady-state entropy production rate can be estimated using the probability fluxes along every edge of the model as^27^
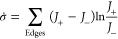
4.1Here, *J*_+_ refers to the flux in the forward direction, and *J*_–_ refers to the flux in the backward direction. For instance, if A and B are two states of a system with reactions between them given by , then *J*_+_ = *k*_1_[A] and *J*_–_ = *k*_2_[B]. Since the entire KaiABC system has been coarse-grained into a minimal Markov system, we underestimate the value of actual entropy production in the entire system.^28^ We use eq 4.1 to estimate the entropy production rate for various values of α, *K*_d0_, and *k*_1_. These results are described in Figures 13 and 14. Of particular note, our results show that σ̇ varies continuously through the transition of the system from a stationary to an oscillatory phase. In the case where the ultrasensitivity parameter *k*_1_ is tuned (Figure 14), the entropy production rate σ̇ is almost a linearly increasing function of *k*_1_. While the entropy production rate σ̇ does indeed increase as oscillations are set up in agreement with previous studies,^21^ and it does indeed improve the overall quality and coherence of oscillation,^29,30^ an analysis focused on just the entropy production rate might miss the important and specific roles played by biophysical mechanisms such as the ultransensitivity and differential affinity in promoting and sustaining robust oscillations.^31^

## V Conclusion

In conclusion, this work elucidates the role played by biophysical mechanisms such as ultrasensitivity and differential affinity in controlling the quality of circadian oscillations. Our minimal theoretical model also provides a route to explain how biochemical circuits can ensure oscillations with constant time periods, even under a range of experimental conditions. Finally, we show that the net rate of energy dissipation is not a very effective order parameter to gauge the quality of oscillations, particularly in regimes where the ultrasensitivity is important, while our work relies on a very minimal abstraction of the KaiABC system. In future work, we hope to adapt these ideas to more complex and complete models of circadian rhythm oscillators.

## Supporting Information Available

Details of the analytical calculations, the numerical simulations, and the methods used (PDF)
