# Supplementary material for: Mechanism for the Generation of Robust Circadian Oscillations through Ultransensitivity and Differential Binding Affinity
Source: J Phys Chem B. 2021 Oct 5;125(40):11179–87. doi: 10.1021/acs.jpcb.1c05915 (PMC8515790; doi:10.1021/acs.jpcb.1c05915)
Supplement: Supplementary file 1 — jp1c05915_si_001.pdf [file jp1c05915_si_001.pdf]

A Mechanism for the Generation of Robust Circadian Oscillations through Ultransensitivity and Differential Binding Affinity: Supplementary information

Agnish Kumar Behera,[1] Clara del Junco,[1] and Suriyanarayanan Vaikuntanathan[1,2]

[1]Department of Chemistry, University of Chicago, Chicago, IL, 60637

[2]The James Franck Institute, University of Chicago, Chicago, IL, 60637*

S1. MODEL DETAILS

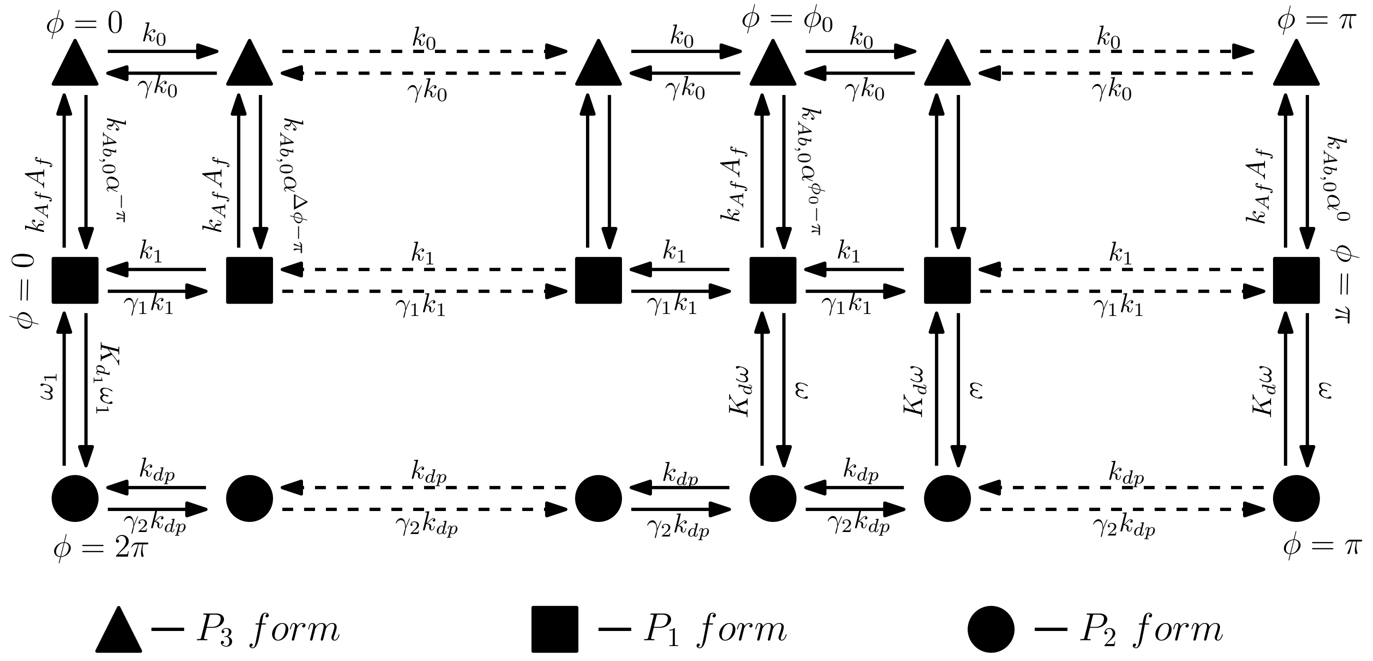

FIG. S1: Each oscillator can exist in one of the three forms P_1, P_2, P_3. The P_1 form corresponds to normal KaiC during daytime and requires KaiA for moving forward in phase. The P_3 form corresponds to the form of KaiC to which KaiA is attached as an assistant molecule, $P_1 + KaiA \longrightarrow P_3$. The P_2 form corresponds to KaiC in it's dephosphorylation phase. The nucleotide bound states, KaiB binding and KaiA sequestration are implicitly assumed in the model. A_f denotes the free KaiA concentration and A_t stands for the total KaiA concentration. Phosphorylation corresponds to phase ϕ, it increases linearly from 0 to 1 as ϕ varies from 0 to π and decreases linearly from 1 to 0 as ϕ goes from π to 2π. Connections between the P_1 states denote the spontaneous dephosphorylation of KaiC subunits in the absence of KaiA. Multiple connections between P_1 and P_2 towards the highly phosphorylated states allows the system to move to the dephosphorylation phase even before all the KaiC is completely phosphorylated. The P_2 states sequester KaiA which corresponds to the fact that during the dephosphorylation phase, KaiB bound KaiC sequesters KaiA and makes it inactive. The master equation for this system is given below.

* Email: svaikunt@uchicago.edu

$$\frac{\partial P_1(\phi)}{\partial t} = \delta(\phi \neq \pi)k_1(P_1(\phi+\Delta\phi) - \gamma_1 P_1(\phi)) + \delta(\phi \neq 0)k_1(\gamma_1 P_1(\phi-\Delta\phi) - P_1(\phi))$$
$$+ \delta(\phi)(\omega_1 P_2(2\pi-\phi) - K_{d1}\omega_1 P_1(\phi))$$
$$+ H(\phi-\phi_0)(K_d \omega P_2(2\pi-\phi) - \omega P_1(\phi)) - k_{Af}A_f P_1(\phi) + k_{Ab,0}\alpha^{\phi-\pi}P_3(\phi) \quad \text{(S1.1)}$$

$$\frac{\partial P_2(\phi)}{\partial t} = \delta(\phi \neq 2\pi)k_{dp}(\gamma_2 P_2(\phi+\Delta\phi) - P_2(\phi)) + \delta(\phi \neq \pi)k_{dp}(P_2(\phi-\Delta\phi) - \gamma_2 P_2(\phi))$$
$$- \delta(\phi-2\pi)(\omega_1 P_2(\phi) - K_{d1}\omega P_1(\phi-2\pi)) + H(2\pi-\phi-\phi_0)(\omega P_1(2\pi-\phi) - \omega P_2(\phi)) \quad \text{(S1.2)}$$

$$\frac{\partial P_3(\phi)}{\partial t} = \delta(\phi \neq \pi)k_0(\gamma P_3(\phi+\Delta\phi) - P_3(\phi)) + \delta(\phi \neq 0)k_0(P_3(\phi-\Delta\phi) - \gamma P_3(\phi))$$
$$+ k_{Af}A_f P_1(\phi) - k_{Ab,0}\alpha^{\phi-\pi}P_3(\phi) \quad \text{(S1.3)}$$

$$A_f = A_t - \sum_0^{\pi} P_3(\phi) - \varepsilon_{seq}\sum_{\pi}^{2\pi} P_2(\phi),\ \varepsilon_{seq} < A_t \quad \text{(S1.4)}$$

$$\frac{dA_f}{dt} = \sum_0^{\pi}(k_{Ab,0}\alpha^{\phi-\pi}P_3(\phi) - k_{Af}A_f P_1(\phi))$$
$$+ \varepsilon_{seq}\sum_{\pi}^{2\pi}(H(2\pi-\phi-\phi_0)\omega P_2(\phi) - K_d \omega P_1(2\pi-\phi)) + \varepsilon(\omega_1 P_2(2\pi) - K_{d1}\omega_1 P_1(0))5 \quad \text{(S1.5)}$$

$$H(\phi) = 1\ for\ \phi \geq 0\ and\ 0\ for\ \phi < 0 \quad \text{(S1.6)}$$

For the sake of convenience, we relabel the P_2 states such that $P_2(2N-j) \Longleftrightarrow P_2(j)$. We also work with the discrete case so we relabel the ϕ using j, where $j = \frac{\phi}{\Delta\phi}$. Relabelling does not change the dynamics. The Fokker-Planck equations in this case are given by,

$$\frac{\partial P_1(j)}{\partial t} = \delta(j \neq N)k_1(P_1(j+1) - \gamma_1 P_1(j)) + \delta(j \neq 0)k_1(\gamma_1 P_1(j-1) - P_1(j))$$
$$+ \delta(j=0)(\omega_1 P_2(j) - K_{d1}\omega_1 P_1(j))$$
$$+ H(j-j_0)(K_d \omega P_2(j) - \omega P_1(j)) - k_{Af}A_f P_1(j) + k_{Ab}\alpha^j P_3(j) \quad \text{(S1.7)}$$

$$\frac{\partial P_2(j)}{\partial t} = \delta(j \neq 0)k_{dp}(\gamma_2 P_2(j-1) - P_2(j)) + \delta(j \neq N)k_{dp}(P_2(j+1) - \gamma_2 P_2(j))$$
$$- \delta(j=0)(\omega_1 P_2(j) - K_{d1}\omega P_1(j)) + H(j-j_0)(\omega P_1(j) - \omega P_2(j)) \quad \text{(S1.8)}$$

$$\frac{\partial P_3(j)}{\partial t} = \delta(j \neq N)k_0(\gamma P_3(j+1) - P_3(j)) + \delta(j \neq 0)k_0(P_3(j-1) - \gamma P_3(j))$$
$$+ k_{Af}A_f P_1(j) - k_{Ab}\alpha^j P_3(j) \quad \text{(S1.9)}$$

$$A_f = A_t - \sum_0^N P_3(j) - \varepsilon_{seq}\sum_0^N P_2(j) \quad \text{(S1.10)}$$

S2. TIME INDEPENDENT STEADY STATE

A. Case I : $k_1 = 0$

We set, $k_1 = 0, j_0 = N, K_{d1} = K_d = K_D$, we also make the following changes in notation $k_{Ab,0}\alpha^{-\pi} = k_{Ab}, k_{dp} = k_2, \alpha -> \alpha^{\frac{\pi}{N}}$. Using this simplification, we can solve for the steady state solution of the system and then use linear stability analysis around the steady state to see how oscillations are set up.

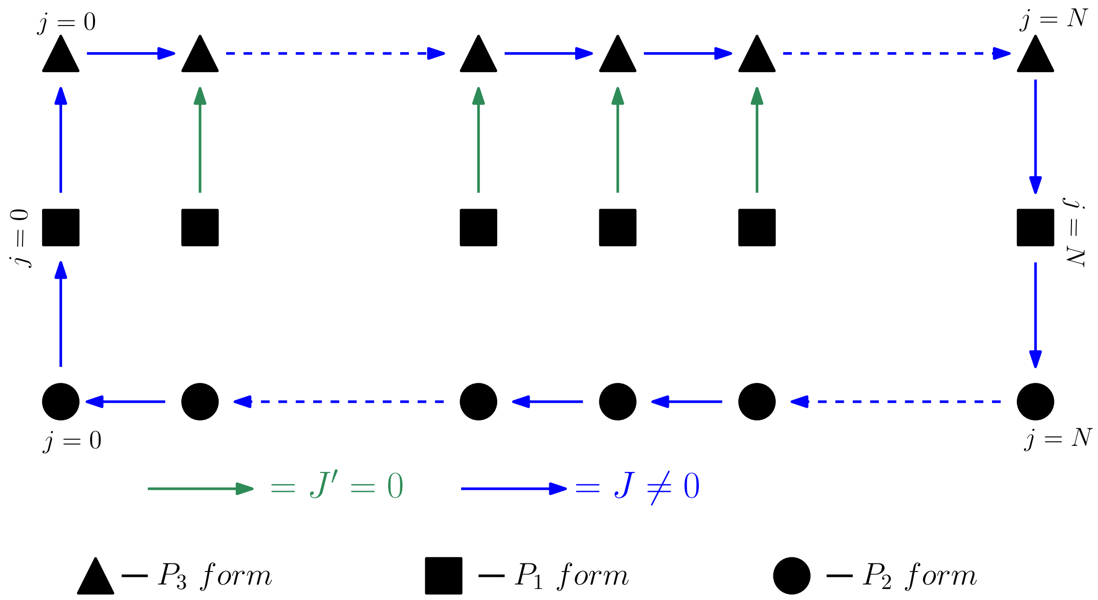

FIG. S2: There is just one flux, J in the entire system. Once we solve for this unique flux J, we can obtain the expressions for the probability distribution of the different states in the system.

$$A_f = A_t - \varepsilon \sum_{j=0}^{N} P_3(j),\; P_3(0) = b,\; P_1(0) = a \tag{S2.1}$$

where a and b are the labels for $P_1(0)$ and $P_3(0)$ respectively. For the $P_1 - P_3$ connection at $j = 0$ and $j = N$ we have,

$$J = k_{Af} A_f P_1(0) - k_{Ab}\alpha^0 P_3(0) = k_{Af} A_f a - k_{Ab} b \tag{S2.2}$$

$$J = k_{Ab}\alpha^N P_3(N) - k_{Af} A_f P_1(N) \tag{S2.3}$$

For other $P_3 - P_3$ connections, we have,

$$J = k_0 P_3(j-1) - \gamma k_0 P_3(j) \tag{S2.4}$$

$$\implies P_3(j) = \frac{1}{\gamma k_0}\left[k_0 P_3(j-1) - J\right] \tag{S2.5}$$

$$\therefore P_3(1) = \frac{1}{\gamma k_0}\left[k_0 b - J\right],\; P_3(j) = \frac{1}{\gamma k_0}\left[\frac{k_0}{\gamma^{j-1}} b - J\left(1 + \frac{1}{\gamma} + \ldots + \frac{1}{\gamma^{j-1}}\right)\right] \tag{S2.6}$$

$$\implies P_3(j) = \frac{1}{\gamma k_0}\left[\frac{k_0}{\gamma^{j-1}} b - J\left(\frac{1 - \frac{1}{\gamma^j}}{1 - \frac{1}{\gamma}}\right)\right] \tag{S2.7}$$

If we look at $P_1 - P_3$ connections in the bulk, we have,

$$J' = 0 \implies k_{Af} A_f P_1(j) = k_{Ab}\alpha^j P_3(j) \;\forall\; j \neq 0, N \tag{S2.8}$$

When we look at the $P_1 - P_2$ connection at $j = 0$, we have,

$$J = \omega_1 P_2(0) - K_D \omega_1 P_1(0) \implies P_2(0) = \frac{1}{\omega_1}\left[J + K_{d1}\omega_1 a\right] = c \tag{S2.9}$$

where c is the label for $P_2(0)$. Similarly for other $P_2 - P_2$ connections we have the following,

$$J = k_2 P_2(j) - \gamma_2 k_2 P_2(j-1) \tag{S2.10}$$

$$\implies P_2(1) = \frac{1}{k_2}\left[J + \gamma_2 k_2 c\right],\ P_2(j) = \frac{1}{k_2}\left[\gamma_2^j k_2 c + J(1 + \gamma_2 + ... + \gamma_2^{j-1})\right] \tag{S2.11}$$

$$\implies P_2(j) = \frac{1}{k_2}\left[\gamma_2^j k_2 c + J\frac{1-\gamma_2^j}{1-\gamma_2}\right] \tag{S2.12}$$

$$\tag{S2.13}$$

Substituting the expressions of $P_1(N)$ (S2.3), $P_2(N)$ (S2.12) and $P_3(N)$ (S2.7) into (S2.14), we get,

$$At\ j = N,\ k_{Ab}\alpha^N P_3(N) - k_{Af}A_f P_1(N) = J = \omega P_1(N) - K_D \omega P_2(N) \tag{S2.14}$$

$$\implies \frac{k_{Ab}\alpha^N}{\gamma^N} - k_{Af}A_f K_d K_{d1}\gamma_2^N a = \left[1 + \frac{k_{Af}A_f}{\omega_1} + \frac{k_{Af}A_f K_d}{k_2}\frac{1-\gamma_2^N}{1-\gamma_2} + \frac{k_{Ab}\alpha^N}{\gamma k_0}\frac{1-\frac{1}{\gamma^N}}{1-\frac{1}{\gamma}} + \frac{k_{Af}A_f K_d \gamma_2^N}{\omega}\right] J \tag{S2.15}$$

$$J = k_{Af}A_f a - k_{Ab} b \tag{S2.16}$$

Since N is large and $\gamma < 1$, the LHS and RHS (S2.15) are dominated by the terms having $\frac{1}{\gamma^N}$. Thus only the 1st term in the LHS and the 3rd term in the RHS of (S2.15) contribute, other terms can be ignored. This leads to an expression for J.

$$\implies J = k_0(1-\gamma)b \tag{S2.17}$$

Substituting this expression for J in (S2.7), (S2.12), (S2.2), (S2.3), (S2.14), we get,

$$\therefore P_3(j) = b \forall j \tag{S2.18}$$

$$P_1(j) = \frac{1}{k_{Af}A_f}\left[k_{Ab}\alpha^j + (\delta_{0,j} - \delta_{N,j})k_0(1-\gamma)\right] b \tag{S2.19}$$

$$P_2(j) = \frac{k_0}{k_2}\left(\frac{1-\gamma}{1-\gamma_2}\right) b + \gamma_2^j\left[k_0(1-\gamma)\left(\frac{1}{\omega_1} - \frac{1}{k_2(1-\gamma_2)}\right) + \frac{K_{d1}\omega}{k_{Af}A_f\omega_1}(k_0(1-\gamma) + k_{Ab})\right] b \tag{S2.20}$$

Now using, $\sum_{j=0}^N (P_1(j) + P_2(j) + P_3(j)) = 1$,

$$\implies \frac{1}{b} = \frac{1}{k_{Af}A_f}\left[k_{Ab}\frac{\alpha^{N+1}-1}{\alpha - 1} + \frac{K_{d1}\omega(k_0(1-\gamma)+k_{Ab})}{(1-\gamma_2)\omega_1}\right] + (N+1)\left(1 + \frac{k_0(1-\gamma)}{k_2(1-\gamma_2)}\right) + \frac{1}{1-\gamma_2}\left(k_0\left(\frac{1-\gamma}{\omega_1}\right) - \frac{k_0(1-\gamma)}{k_2(1-\gamma_2)}\right) \tag{S2.21}$$

$A_f = A_t - \varepsilon\sum_{j=0}^N P_3(j) - \varepsilon_{seq}\sum_{j=0}^N P_3(j) = A_t + f - gb$, where f and g are constants. Setting $\varepsilon_{seq} = 0$, we get, $A_f = A_t - (N+1)b$ and we need to solve a quadratic equation to find the probabilities which determine the steady state.

B. Case II : $k_1 \neq 0$

The case with $k_1 \neq 0$ is challenging to solve. Unlike the previous case, where only a single flux existed in the entire system, in this case there will be many fluxes in the system.

In order to obtain the rough form of solution for P_1, P_2 and P_3 states, we make some assumptions which are supported by numerical observations. We also go the continuum limit where the discrete master equations describing the system become a set of coupled PDE's. The boundaries for our problem are $x = 0$ and $x = x_0$. x_0 is the point where the $P_1 - P_2$ connections start. Numerically it is observed that at the steady state, the probability density in the states beyond x_0 is negligible compared to the ones before it. Thus we set it as our boundary. We solve the problem for the states in the bulk and then impose certain conditions

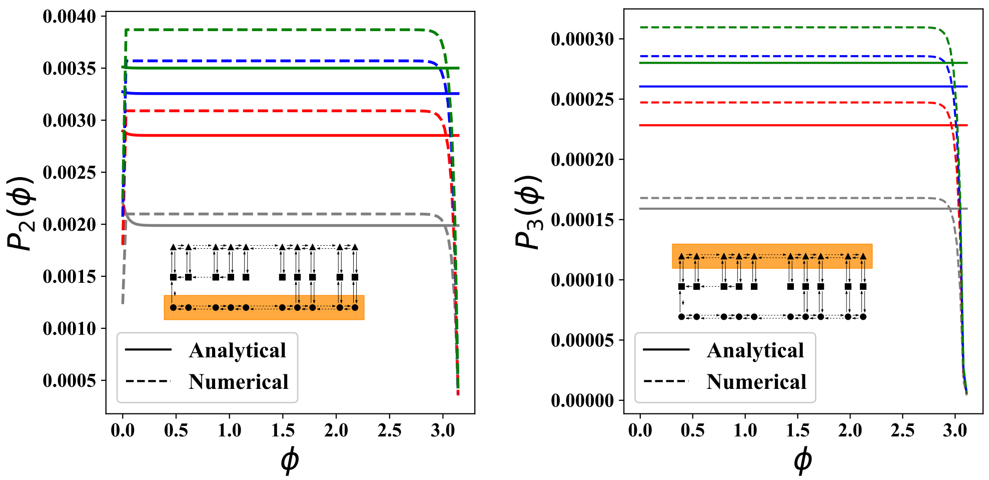

(a) Comparison between numerical and analytical results for time-independent solution of P_2 states. The figure in inset is a representation of the Markov State network with the P_1 states highlighted.

(b) Comparison between numerical and analytical results for time-independent solution of P_3 states. The figure in inset is a representation of the Markov State network with the P_1 states highlighted.

FIG. S3: In the main figures, *grey* corresponds to $\alpha = 2$, *red* to $\alpha = 4$, *blue* to $\alpha = 6$ and *green* to $\alpha = 8$

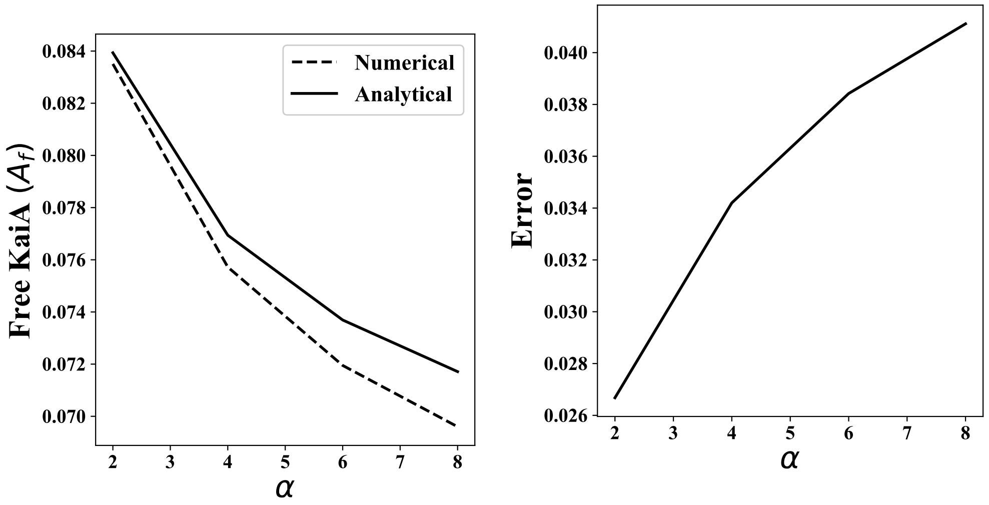

(a) Comparison between numerical and analytical results for free KaiA concentration.

(b) Comparison of error between numerical and analytical results. Error = $1 - \left| \frac{\vec{P^s}_{numerical}\vec{P^s}_{analytical}}{\vec{P^s}_{numerical}\vec{P^s}_{numerical}} \right|$

FIG. S4: Comparison of free KaiA concentration and error between the analytical data and numerical data.

such that the boundary conditions are satisfied.

$$\frac{\partial P_1(x)}{\partial t} = \delta(x \neq N)k_1(P_1(x+\Delta x) - \gamma_1 P_1(x)) + \delta(x \neq 0)k_1(\gamma_1 P_1(x-\Delta x) - P_1(x))$$
$$+\delta(x=0)(\omega_1 P_2(x) - K_{d1}\omega_1 P_1(x))$$
$$+H(x-x_0)(K_d\omega P_2(x) - \omega P_1(x)) - k_{Af}A_f P_1(j) + k_{Ab}\alpha^x P_3(j) \quad \text{(S2.22)}$$
$$\text{(S2.23)}$$

and so on and so forth for P_2 and P_3 states. Using Taylor expansion, $P(x+\Delta x) = P(x) + \frac{\partial P(x)}{\partial x}\Delta x + \frac{1}{2}\frac{\partial^2 P(x)}{\partial x^2}\Delta x^2 + O(\Delta x^3)$, we get,

$$\frac{\partial P_1(x)}{\partial t} = k_{1c}\frac{\partial P_1(x)}{\partial x} + k_{1d}\frac{\partial^2 P_1(x)}{\partial x^2} - k_{Af}A_f P_1(x) + k_{Ab}\alpha^x P_3(x) \tag{S2.24}$$

$$\frac{\partial P_2(x)}{\partial t} = k_{2c}\frac{\partial P_2(x)}{\partial x} + k_{2d}\frac{\partial^2 P_2(x)}{\partial x^2} \tag{S2.25}$$

$$\frac{\partial P_3(x)}{\partial t} = k_{3c}\frac{\partial P_3(x)}{\partial x} + k_{3d}\frac{\partial^2 P_3(x)}{\partial x^2} + k_{Af}A_f P_1(x) - k_{Ab}\alpha^x P_3(x) \tag{S2.26}$$

$$k_{1c} = k_1(1-\gamma_1)\Delta x,\ k_{2c} = k_{dp}(1-\gamma_2)\Delta x,\ k_{3c} = -k_0(1-\gamma)\Delta x \tag{S2.27}$$

$$k_{1d} = \frac{1}{2}k_1(1+\gamma_1)\Delta x^2,\ k_{2d} = \frac{1}{2}k_{dp}(1+\gamma_2)\Delta x^2,\ k_{3d} = \frac{1}{2}k_0(1+\gamma)\Delta x^2 \tag{S2.28}$$

We begin with the ansatz that when k_1 is increased from 0 to a very small number gradually, the changes in the form of the probability distribution will not change drastically. Keeping this in mind we make the assumption, $k_{Af}A_f P_1(x) \approx k_{Ab}\alpha^x P_3(x) \forall x \in Bulk$. This assumption has been inspired by our solution for the $k_1 = 0$ case and also supported by numerical observations. It can be better written as,

$$P_1(x) = \frac{K_{d0}}{A_f}\alpha^x P_3(x) \tag{S2.29}$$

, where $K_{d0} = \frac{k_{Ab}}{k_{Af}}$. Thus, we have,

$$\partial_x P_1(x) = \frac{K_{d0}}{A_f}\alpha^x\left[ln(\alpha) + \partial_x P_3(x)\right] \tag{S2.30}$$

$$\partial_x^2 P_1(x) = \frac{K_{d0}}{A_f}\alpha^x\left[(ln(\alpha))^2 P_3(x) + 2ln(\alpha)\partial_x P_3(x) + \partial_x^2 P_3(x)\right] \tag{S2.31}$$

Adding the evolution equations for P_1 and P_3, in the bulk, and substituting the approximation (S2.29) we get,

$$\begin{aligned}\partial_t(P_1(x)+P_3(x)) &= \frac{K_{d0}}{A_f}\alpha^x ln(\alpha)[k_{1c} + k_{1d}ln(\alpha)]P_3(x)\\ &+ \left[k_{3c} + \frac{K_{d0}}{A_f}\alpha^x(k_{1c} + k_{1d}ln(\alpha))\right]\partial_x P_3(x) +\\ &+ \left[k_{3d} + \frac{K_{d0}}{A_f}\alpha^x k_{1d}\right]\partial_x^2 P_3(x)\end{aligned} \tag{S2.32}$$

At steady state, $\partial_t P_1(x) = 0 = \partial_t P_3(x)\ \forall x$. For $k_1 = 0$, we have $k_{1c} = 0 = k_{1d}$. Thus we have the simple ODE,

$$k_{1c}\partial_x P_3(x) + k_{3d}\partial_x^2 P_3(x) = 0$$

This can have constant solutions for $P_3(x)$ and this is exactly what we have in the case when $k_1 = 0$ (S2.18). The presence of k_1 adds an extra term dependent on P_3 and due to this term we cannot have constant solutions for $P_3(x)$ (unless the constant solution is $P_3(x) = 0 \forall x$). Under the assumption that the form of $P_3(x)$ does not deviate significantly from the solution when $k_1 = 0$ ($P_3(x)$ = b = constant), we can ignore terms containing $\partial_x^2 P_3(x)$. We also have $k_{3d} << k_{3c}$, $k_{1d} << k_{1c}$. Using this we can ignore terms containing k_{1d} and k_{3d}. Thus we finally arrive at the equation,

$$\frac{K_{d0}}{A_f}\alpha^x ln(\alpha)k_{1c}P_3(x) + \left[k_{3c} + \frac{K_{d0}}{A_f}\alpha^x k_{1c}\right]\partial_x P_3(x) = 0 \tag{S2.33}$$

$$\implies P_3(x) = P_3(x')\frac{|B + A\alpha^{x'}|}{|B + A\alpha^x|} \tag{S2.34}$$

$$B = k_{3c},\ A = \frac{K_{d0}}{A_f}k_{1c} \tag{S2.35}$$

Beyond the boundary at $x = x_0$, the network is constructed in such a way that it either drives the probabilities into the P_2 states which are further driven towards the boundary at x_0 from right or it drives the probabilities towards the boundary at x_0 in the P_1

states.

Thus we can safely assume that probability of the finding a state beyond the boundary at x_0 is close to 0. This is confirmed by numerical results. Now we can focus our entire attention to the region, $x \in [0, x_0]$. By using conservation of flux we can find the probabilities of all the other states.

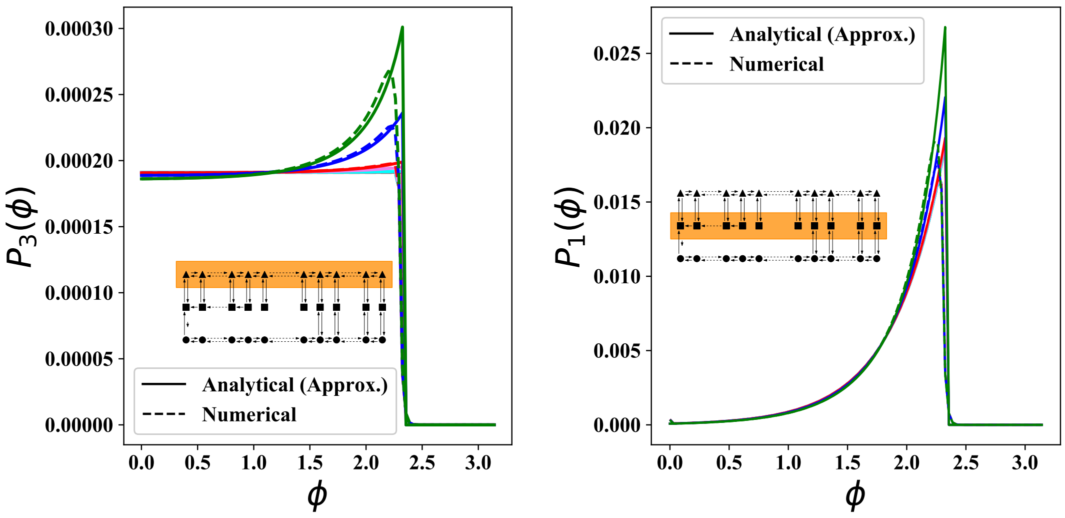

(a) Comparison between numerical and analytical results for time-independent solution of P_3 states. The figure in inset is a representation of the Markov State network with the P_3 states highlighted.

(b) Comparison between numerical and analytical results for time-independent solution of P_1 states. The figure in inset is a representation of the Markov State network with the P_1 states highlighted.

FIG. S5: In the main figures, *grey* corresponds to $k_1 = 0$, *cyan* to $k_1 = 10^{-4}$, *violet* to $k_1 = 5 \times 10^{-4}$, *red* to $k_1 = 10^{-3}$, *blue* to $k_1 = 5 \times 10^{-3}$, *green* to $k_1 = 10^{-2}$.

C. Calculating Time-Independent Steady State numerically

As mentioned earlier, when $k_1 \neq 0$ and there are more than one connections between P_1 and P_2 states i.e. $j_{0}, x_0 \neq N$, we have multiple fluxes in the system. Nevertheless, we can still find the steady-state time-independent solution for $\vec{P}$ irrespective of whether it is stable or not. An iterative procedure is adopted. The first step in this procedure is to find the free KaiA concentration in the system. In the following paragraph the procedure is described. The set of FPE's that describe the evolution of $\vec{P}$ can be expressed as, $\frac{\partial \vec{P}}{\partial t} = W(\vec{P})\vec{P}$, where $\vec{P}$ is a vector of length $3N+3$. The first N+1 elements would correspond to P_1 form, the next N+1 elements would correspond to P_2 form and the last N+1 elements would correspond to P_3 form. The rate matrix, W is function of the probabilities due to the presence of the A_f term which makes the entire thing non-linear. Now if we succeed in finding the free KaiA concentration at steady state, then substituting it back into W would make it a linear system to solve, and then $\frac{\partial \vec{P}}{\partial t} = W\vec{P}$. We can find A_f^s (free KaiA at steady state) using an iterative procedure as follows:

1. Initialize $A_f = A_t$ for the first run and form the rate matrix, W.

2. At steady state, $\frac{\partial \vec{P}}{\partial t} = 0 = W\vec{P}$. Compute the eigenvector corresponding to the nullspace of W and call it $\vec{v_0}$.

3. Compute $A_f^{test} = A_t - \varepsilon \sum_{i=2N+2}^{3N+2} v_0(i) - \varepsilon_{seq} \sum_{i=N+1}^{2N+1} v_0(i)$

4. If $A_f^{test} < 0$, it would be unphysical. So set, $A_f^{new} = \frac{A_f^{old}}{2}$, else set $A_f^{new} = A_f^{old} + \delta(A_f^{test} - A_f^{old})$ where δ is some appropriate step size.

5. Repeat this procedure until convergence i.e. $\frac{|A_f^{old} - A_f^{test}|}{A_f^{old}} < Tolerance$

Once we have A_f^s we can find $\vec{P^s}$.

S3. LINEAR STABILITY ANALYSIS

We perturb around the steady state distribution, $\vec{P^s}$. Say, $P_k(j) = P^s_k(j) + \delta\eta_k(j)$, $k = 1,2,3$ and $j = 0,...,N$. By conservation of probability, we have $\sum_{k,j}\eta_k(j) = 0$. Substituting $P_k(j)$ in the differential equations, lead us to the evolution equations for $\eta_k(j)$.

$$\begin{aligned}\frac{\partial\eta_1(j)}{\partial t} &= \delta(j\neq N)k_1(\eta_1(j+1)-\gamma_1\eta_1(j)) + \delta(j\neq 0)k_1(\gamma_1\eta_1(j-1)-\eta_1(j))\\ &+\delta(j=0)(\omega_1\eta_2(j)-K_{d1}\omega_1\eta_1(j))\\ &+H(j-j_0)(K_d\omega\eta_2(j)-\omega\eta_1(j)) - k_{Af}A^s_f\eta_1(j)+k_{Ab}\alpha^j\eta_3(j)\\ &+\varepsilon k_{Af}P^s_1(j)\sum_{i=0}^{N}\eta_3(i)+\varepsilon_{seq}k_{Af}P^s_1(j)\sum_{i=0}^{N}\eta_2(i)+O(\delta) \end{aligned} \tag{S3.1}$$

$$\begin{aligned}\frac{\partial\eta_2(j)}{\partial t} &= \delta(j\neq 0)k_{dp}(\gamma_2\eta_2(j-1)-\eta_2(j)) + \delta(j\neq N)k_{dp}(\eta_2(j+1)-\gamma_2\eta_2(j))\\ &-\delta(j=0)(\omega_1\eta_2(j)-K_{d1}\omega\eta_1(j))\\ &+H(j-j_0)(\omega\eta_1(j)-\omega\eta_2(j)) \end{aligned} \tag{S3.2}$$

$$\begin{aligned}\frac{\partial\eta_3(j)}{\partial t} &= \delta(j\neq N)k_0(\gamma\eta_3(j+1)-\eta_3(j)) + \delta(j\neq 0)k_0(\eta_3(j-1)-\gamma\eta_3(j))\\ &+k_{Af}A^s_f\eta_1(j)-k_{Ab}\alpha^j\eta_3(j)\\ &-\varepsilon k_{Af}P^s_1(j)\sum_{i=0}^{N}\eta_3(i)-\varepsilon_{seq}k_{Af}P^s_1(j)\sum_{i=0}^{N}\eta_2(i)+O(\delta) \end{aligned} \tag{S3.3}$$

Notice the additional terms in evolution of $\vec{\eta}_1$ and $\vec{\eta}_3$ which are directly dependent on ε and ε_{seq}. The entire thing can be expressed as $\frac{\partial\vec{\eta}}{\partial t} = \tilde{\mathbf{W}}\vec{\eta} = (\mathbf{W}+\mathbf{W}')\vec{\eta}$. The entire matrix $\tilde{W}$ can be broken into 9 parts, each representing interactions between different types of states as shown in (S3.5). The interesting blocks in the W-matrix are the $\eta_3-\eta_3$ (S3.6) and $\eta_3-\eta_2$ (S3.7) blocks which contain most of the terms arising due to nonlinearities.

In short, a linear stability analysis can be performed around the steady state of the system, $\vec{P^s}$, which yields upto first order,

$$\frac{\partial\vec{\eta}}{\partial t} = [\mathbf{W}(\tilde{\mathbf{P}}^{\mathbf{s}}) + \nabla_{\vec{P}}\mathbf{W}(\tilde{\mathbf{P}})\vec{P}|_{\vec{P^s}}]\vec{\eta} = [\mathbf{W}(\tilde{\mathbf{P}}^{\mathbf{s}})+\mathbf{W}'(\tilde{\mathbf{P}}^{\mathbf{s}})]\vec{\eta} = \tilde{\mathbf{W}}\vec{\eta} \tag{S3.4}$$

where η is the vector of perturbation (see Section S3 for a detailed expression). We work in a regime where the %ATP i.e. K_{d0} in our model plays a major role in deciding whether oscillations take place or not. The initial condition for every simulation is set to $P_1(0) = 1$ at t=0. This corresponds to starting all reactions with all the KaiC in the unphosphorylated and ADP bound form. The simulations are allowed to run for some time in order to reach either a time-independent or a time-dependent steady state behaviour. Now let us look at the $\mathbf{W}$ matrix. It can be broken into 9 blocks,

$$\tilde{W} = \left(\begin{array}{c|c|c} W_{11} & W_{12} & W_{13}\\ \hline W_{21} & W_{22} & W_{23}\\ \hline W_{31} & W_{32} & W_{33}\end{array}\right) \tag{S3.5}$$

$$W_{33} = \begin{pmatrix} -k_{Ab}\alpha^0-\varepsilon k_{Af}P^s_1(0) & -\varepsilon k_{Af}P^s_1(0) & -\varepsilon k_{Af}P^s_1(0) & \dots & -\varepsilon k_{Af}P^s_1(0)\\ -\varepsilon k_{Af}P^s_1(1) & -k_{Ab}\alpha^1-\varepsilon k_{Af}P^s_1(1) & -\varepsilon k_{Af}P^s_1(1) & \dots & -\varepsilon k_{Af}P^s_1(1)\\ \vdots & \dots & \ddots & \dots & \vdots\\ -\varepsilon k_{Af}P^s_1(N-1) & \dots & -\varepsilon k_{Af}P^s_1(N-1) & -k_{Ab}\alpha^{N-1}-\varepsilon k_{Af}P^s_1(N-1) & -\varepsilon k_{Af}P^s_1(N-1)\\ -\varepsilon k_{Af}P^s_1(N) & \dots & -\varepsilon k_{Af}P^s_1(N) & -\varepsilon k_{Af}P^s_1(N) & -k_{Ab}\alpha^N-\varepsilon k_{Af}P^s_1(N)\end{pmatrix} \tag{S3.6}$$

$$W_{32} = \begin{pmatrix} -\varepsilon_{seq}k_{Af}P_1^s(0) & -\varepsilon_{seq}k_{Af}P_1^s(0) & -\varepsilon_{seq}k_{Af}P_1^s(0) & \dots & -\varepsilon_{seq}k_{Af}P_1^s(0) \\ -\varepsilon_{seq}k_{Af}P_1^s(1) & -\varepsilon_{seq}k_{Af}P_1^s(1) & -\varepsilon_{seq}k_{Af}P_1^s(1) & \dots & -\varepsilon_{seq}k_{Af}P_1^s(1) \\ \vdots & \dots & \ddots & \dots & \vdots \\ -\varepsilon_{seq}k_{Af}P_1^s(N-1) & \dots & -\varepsilon_{seq}k_{Af}P_1^s(N-1) & -\varepsilon_{seq}k_{Af}P_1^s(N-1) & -\varepsilon_{seq}k_{Af}P_1^s(N-1) \\ -\varepsilon_{seq}k_{Af}P_1^s(N) & \dots & -\varepsilon_{seq}k_{Af}P_1^s(N) & -\varepsilon_{seq}k_{Af}P_1^s(N) & -\varepsilon_{seq}k_{Af}P_1^s(N) \end{pmatrix} \tag{S3.7}$$

To make things simpler let us look at just the $\mathbf{W}'$ matrix elements,

$$\mathbf{W}' = \left(\begin{array}{c|c|c} W'_{11} & W'_{12} & W'_{13} \\ \hline W'_{21} & W'_{22} & W'_{23} \\ \hline W'_{31} & W'_{32} & W'_{33} \end{array}\right) \tag{S3.8}$$

$$W'_{33} = \begin{pmatrix} -\varepsilon k_{Af}P_1^s(0) & -\varepsilon k_{Af}P_1^s(0) & \dots & -\varepsilon k_{Af}P_1^s(0) \\ -\varepsilon k_{Af}P_1^s(1) & -\varepsilon k_{Af}P_1^s(1) & \dots & -\varepsilon k_{Af}P_1^s(1) \\ \vdots & \dots & \ddots & \vdots \\ -\varepsilon k_{Af}P_1^s(N) & \dots & -\varepsilon k_{Af}P_1^s(N) & -\varepsilon k_{Af}P_1^s(N) \end{pmatrix} \tag{S3.9}$$

$$W'_{32} = \begin{pmatrix} -\varepsilon_{seq}k_{Af}P_1^s(0) & -\varepsilon_{seq}k_{Af}P_1^s(0) & \dots & -\varepsilon_{seq}k_{Af}P_1^s(0) \\ -\varepsilon_{seq}k_{Af}P_1^s(1) & -\varepsilon_{seq}k_{Af}P_1^s(1) & \dots & -\varepsilon_{seq}k_{Af}P_1^s(1) \\ \vdots & \dots & \ddots & \vdots \\ -\varepsilon_{seq}k_{Af}P_1^s(N) & \dots & -\varepsilon_{seq}k_{Af}P_1^s(N) & -\varepsilon_{seq}k_{Af}P_1^s(N) \end{pmatrix} \tag{S3.10}$$

Calculating the eigenvalues of W tells us about the stability of the steady state. Presence of +ve eigenvalues would indicate that the steady state is unstable and that a time-dependent steady state is present in the system.This would give rise to oscillations.

A. Origin of Instability

Increasing α leads to accumulation of probability density near the higher phosphorylated region of P_1 and P_3 states. Eventually, this leads to an instability. The oscillatory state is stable because higher α provides coherence to the wavepacket i.e. the phosphorylation wavepacket has a narrow width as it moves across the different states [1].

One simple way to understand the emergence of oscillations with increasing k_1 is through the Gershgorin circle theorem. The Gershgorin circle theorem provides us a way to estimate the location of the eigenvalues of any square matrix. Simply put, it says that the for any square matrix W, if we construct the pair (W_{ii}, R_i), where $R_i = \sum_{j,j\neq i}|W_{ji}|$, then all the eigenvalues of W lie in the union of circles with radii R_i and centred at W_{ii}. The onset of instability means the presence of +ve eigenvalues in the W matrix as mentioned before. In a normal rate matrix, all the diagonal entries are -ve and the off-diagonal entries are +ve in a way such that sum of all element in each column is 0. Gershgorin theorem can be easily applied to this system and it can be seen that the eigenvalues will always have to be -ve (or 0). But in our case, the matrix W does have -ve off-diagonal elements, for instance $-\varepsilon k_{Af}P_1^s(j)\eta_3(i), i \neq j$ term in the evolution of $\eta_3(j)$. All such terms which are -ve in the off-diagonal position have P_1^s. It is the presence of these terms which extend the Gershgorin circles into the positive half of the plane. So, our chances of obtaining a positive eigenvalue increases if we have higher $P_1^s(j)\forall j$. Now it remains to show that as K_{d0} increases, P_1^s either decreases or stays unchanged and when k_1 increases, P_1^s increases.

From (S2.33) we know the forms of the solution for $k_1 \neq 0$. For a moment let us take A_f to be fixed. This assumption is justified in the limit when k_1 is very small and there is not much change in the value of A_f derived in the $k_1 = 0$ case. For this fixed value of A_f let us consider two cases, the first when $k_1 = 0$ and the second when $k_1 \neq 0$. Fixed A_f implies that $\sum P_3(i) = constant = c$. Let us denote the functional form for $P_3(x)$ as f(x) for case I and g(x) for case II. As we have shown previously f(x) is a constant function and g(x) is a strictly increasing function. $\sum_x(f(x) - g(x)) = 0$ and the fact that g(x) is strictly increasing implies that f(x) and g(x) have a single point where they cross each other i.e. $f(x) > g(x)$ for $x < x_c$, $f(x) = g(x)$ at $x = x_c$ and $f(x) < g(x)$ for $x > x_c$, where x_c is the point of crossover. Now $\sum_x(P_1^{II}(x) - P_1^{I}(x)) = \frac{K_{d0}}{A_f}\sum_x \alpha^x(g(x) - f(x))$. This is a polynomial in α with a single sign change in the coefficients at $x = x_c$, with 1 as a root and with the leading term $(g(x_N) - f(x_N))$ to be positive. Thus from Decartes rule for change in signs we can can say that 1 is the only positive root of the polynomial and thus $\sum_x \alpha^x(g(x) - f(x)) > 0 \; \forall \alpha > 1$. Thus we can say that increasing k_1 increases $\sum_x P_1(x)$. This in turn affects the radii of the Gershgorin circles and thus the possibility of having an eigenvalue in the positive half of the complex plane increases with increasing k_1.

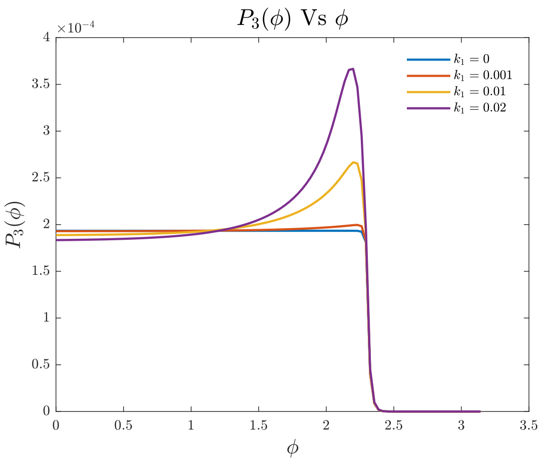

(a) For $k_1 = 0$ we have a constant solution for $P_3(\phi)$. For $k_1 \neq 0$, we have a solution of the form (S2.34).

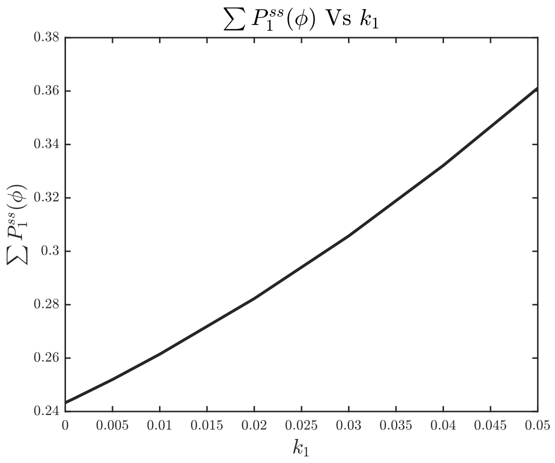

(b) This graph shows how the total probability in the P_1 form at steady state increases as a function of k_1.

FIG. S6: Origin of Instability

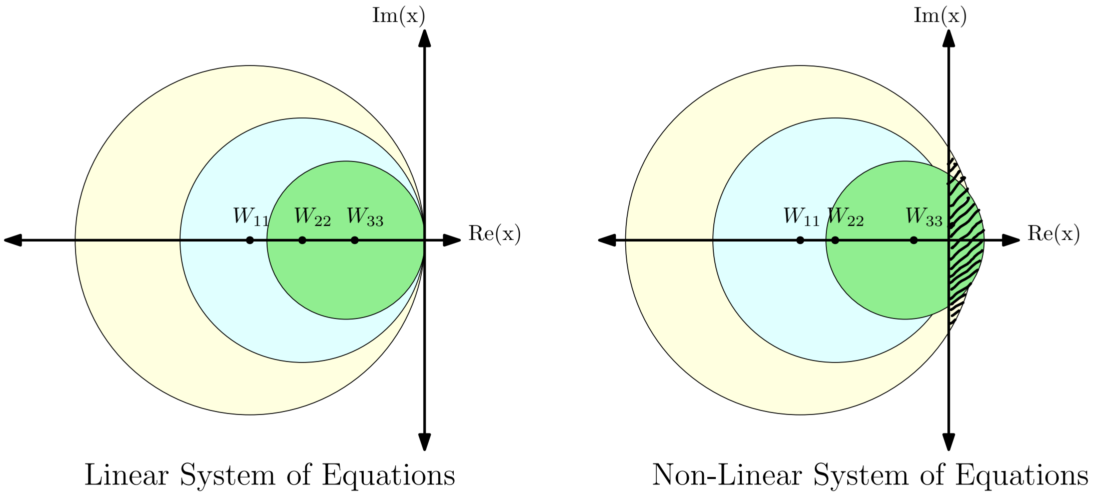

FIG. S7: All the eigenvalues lie in within the area of the union of all circles. In The linear case, the union of all circles lies completely in the left half of the Argand plane. Thus no eigenvalues are possible which have a +ve real part. In the nonlinear case, due to the -ve off diagonal terms, there is a possibility to have eigenvalues with +ve real part. If the shaded reagion has a greater area then chances of getting an instability increases.

S4. FIRST PASSAGE ANALYSIS

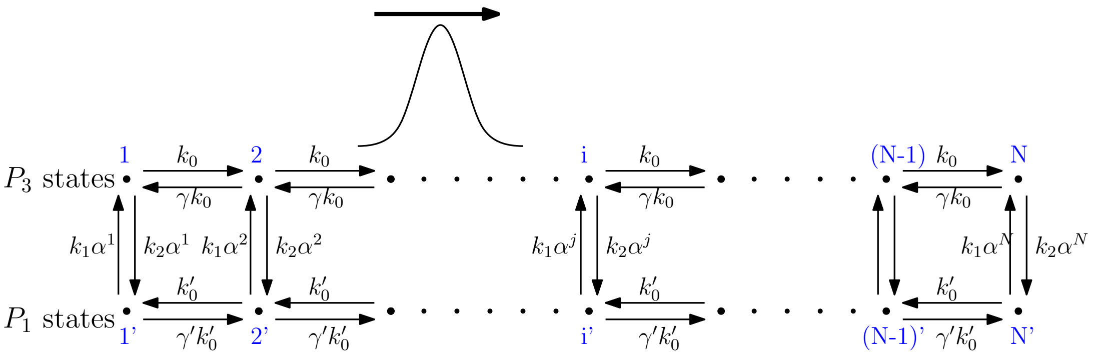

FIG. S8: The wave of phosphorylation moves across the P_1 and P_3 states. The movement of the wavepacket can be realized as a particle hopping between sites labelled with indices i (for P_3 states) and i' (for P_1 states)

In the notation used to solve the Fokker Planck equations, $k_1\alpha^x$ would correspond to $k_{A_f}A_f$, $k_2\alpha^x$ would correspond to $k_{Ab0}\alpha^{x-\pi}$, k_0' to k_1 and γ_0' to γ_1. It is observed as the wavepacket moves, the free KaiA concentration changes with time as a function of α as, $A_f = A_{f0}\alpha^x$ where x is the position of the tip of the wavepacket.

Let T_n denote the time taken for the particle to reach the end for the first time, starting from the nth position.

$$T_1 = \frac{k_2\alpha}{k_2\alpha + k_0}\left(T_{1'} + \frac{1}{k_2\alpha}\right) + \frac{k_0}{k_0 + k_2\alpha}\left(T_2 + \frac{1}{k_0}\right) \tag{S4.1}$$

$$T_{1'} = \frac{k_1\alpha}{k_1\alpha + \gamma' k_0'}\left(T_1 + \frac{1}{k_1\alpha}\right) + \frac{\gamma' k_0'}{k_1\alpha + \gamma' k_0'}\left(T_2' + \frac{1}{\gamma' k_0'}\right) \tag{S4.2}$$

$$T_2 = \frac{k_2\alpha^2}{k_0(1+\gamma) + k_2\alpha^2}\left(T_{2'} + \frac{1}{k_2\alpha^2}\right) + \frac{k_0}{k_0(1+\gamma) + k_2\alpha^2}\left(T_3 + \frac{1}{k_0}\right) + \frac{\gamma k_0}{k_0(1+\gamma) + k_2\alpha^2}\left(T_1 + \frac{1}{\gamma k_0}\right) \tag{S4.3}$$

$$T_{2'} = \frac{k_1\alpha^2}{k_0'(1+\gamma') + k_1\alpha^2}\left(T_2 + \frac{1}{k_1\alpha^2}\right) + \frac{\gamma' k_0'}{k_0'(1+\gamma') + k_1\alpha^2}\left(T_{3'} + \frac{1}{\gamma' k_0'}\right) + \frac{k_0'}{k_0'(1+\gamma') + k_1\alpha^2}\left(T_{1'} + \frac{1}{k_0'}\right) \tag{S4.4}$$

and so on and so forth. Define:

$$s_j = k_0(1+\gamma) + k_2\alpha^j \;\; p_j = \frac{k_2\alpha^j}{s_j} \;\; q_j = \frac{k_0}{s_j} \;\; r_j = \frac{\gamma k_0}{s_j} \tag{S4.5}$$

and similarly for the indices j'. Using these definitions, we have,

$$T_j = p_j T_{j'} + q_j T_{j+1} + r_j T_{j-1} + 3s_j^{-1} \tag{S4.6}$$

$$T_{j'} = p_{j'} T_j + r_{j'} T_{j'+1} + q_{j'} T_{j'-1} + 3s_j^{-1} \tag{S4.7}$$

$$\implies T_j = T_{j'} + \frac{3}{p_j s_j} + \frac{q_j - r_j}{p_j}\frac{\partial T_j}{\partial j} \tag{S4.8}$$

$$T_{j'} = T_j + \frac{3}{p_{j'} s_{j'}} + \frac{r_j - q_j}{p_j}\frac{\partial T_{j'}}{\partial j'} \tag{S4.9}$$

Eliminating T' and relabelling j by x, we get,

$$\frac{(1-\gamma)(1-\gamma')k_0k_0'}{k_1k_2\alpha^x}\frac{\partial^2 T}{\partial x^2} + \left[\frac{(1-\gamma)k_0}{k_2} - \frac{(1-\gamma')k_0'}{k_1}\right]\frac{\partial T}{\partial x} + 3\left[\frac{1}{k_1} + \frac{1}{k_2} - \frac{(1-\gamma')k_0' ln\alpha}{k_1k_2\alpha^x}\right] = 0 \tag{S4.10}$$

Assuming that T is linear in x for small k_0', the first term can be neglected. The solution of T(x) is given as,

$$AT(x) = -\frac{C}{ln\alpha}(1-\alpha^{-L}) - B(x-L) \tag{S4.11}$$

(S4.12)

where, $A = \frac{(1-\gamma)k_0}{k_2} - \frac{(1-\gamma')k_0'}{k_1}$, $B = \frac{1}{k_1} + \frac{1}{k_2}$ and $C = \frac{(1-\gamma')k_0' ln\alpha}{k_1 k_2}$ The velocity of the wave packet is given by, $v = \frac{L}{T(0)}$. Putting in all the values, we get,

$$v = \frac{1}{3}\frac{(1-\gamma)k_0 - \frac{k_2}{k_1}(1-\gamma')k_0'}{1+\frac{k_2}{k_1} - \frac{k_0'}{k_1}(1-\gamma')ln\alpha} \approx \frac{1}{3}\frac{(1-\gamma)k_0 - \frac{k_2}{k_1}(1-\gamma')k_0'}{1+\frac{k_2}{k_1}} \tag{S4.13}$$

In the regime where we have oscillations, $k_0' << k_1$. Thus the second term in the denominator can be ignored. We have, $\frac{k_2}{k_1} = \frac{k_{Ab0}\alpha^{-\pi}}{k_{Af}A_{f0}} = \frac{K_{d0}}{\alpha^{\pi}A_{f0}}$. This expression shows that with increase in K_{d0}, the velocity of the wavepacket decreases.

S5. PARAMETERS

k_0	2.5	γ	0.5	ω	10^{-1}
k_1	$0{\cdot}10^{-2}$	γ_1	10^{-2}	K_d	10^{-1}
k_{dp}	$2{\cdot}10^{-1}$	γ_{dp}	$5\cdot10^{-2}$	ω_1	1
$k_{Ab,0}$	10^3	K_{d0}	1-11	K_{d1}	10^{-1}
α	10	A_t	0.1	ε_{seq}	0

TABLE S1: Parameter Values for comparing Analytical prediction and Numerical Simulation in the $k_1 = 0$ case, i.e. when ultrasensitivity is absent.

k_0	2.5	γ	0.08	ω	10^{-1}
k_1	$1\text{-}5{\cdot}10^{-2}$	γ_1	10^{-2}	K_d	10^{-2}
k_{dp}	$5{\cdot}10^{-2}$	γ_{dp}	$5\cdot10^{-2}$	ω_1	10^{-2}
$k_{Ab,0}$	10^3	K_{d0}	1-11	K_{d1}	10^{-2}
α	10	A_t	0.1	ε_{seq}	0.1

TABLE S2: Parameter Values for Numerical simulations in the $k_1 \neq 0$ case, i.e. when ultrasensitivity is present.

All numerical simulations were performed using ODE15s function of MATLAB. In all our simulations, the value of N was 100, i.e. there were 101 states of each type, P_1, P_2 and P_3.

[1] Dongliang Zhang, Yuansheng Cao, Qi Ouyang, and Yuhai Tu. The energy cost and optimal design for synchronization of coupled molecular oscillators. *Nature Physics*, 16(1):95–100, 2020.
